# Ancient Mitogenomes Reveal the Origins and Genetic Structure of the Neolithic Shimao Population in Northern China

**DOI:** 10.3389/fgene.2022.909267

**Published:** 2022-05-27

**Authors:** Jiayang Xue, Wenjun Wang, Jing Shao, Xiangming Dai, Zhouyong Sun, Jacob D. Gardner, Liang Chen, Xiaoning Guo, Nan Di, Xuesong Pei, Xiaohong Wu, Ganyu Zhang, Can Cui, Peng Cao, Feng Liu, Qingyan Dai, Xiaotian Feng, Ruowei Yang, Wanjing Ping, Lizhao Zhang, Nu He, Qiaomei Fu

**Affiliations:** ^1^ Key Laboratory of Vertebrate Evolution and Human Origins, Institute of Vertebrate Paleontology and Paleoanthropology, Center for Excellence in Life and Paleoenvironment, Chinese Academy of Sciences, Beijing, China; ^2^ University of Chinese Academy of Sciences, Beijing, China; ^3^ Science and Technology Archaeology, National Centre for Archaeology, Beijing, China; ^4^ Shaanxi Academy of Archaeology, Xi’an, China; ^5^ Archaeology Institute of National Museum of China, Beijing, China; ^6^ School of Cultural Heritage, Northwest University, Xi’an, China; ^7^ School of Archaeology and Museology, Peking University, Beijing, China; ^8^ Institute of Archaeology, Chinese Academy of Social Sciences, Beijing, China; ^9^ Shanghai Qi Zhi Institute, Shanghai, China

**Keywords:** Shimao, mitochondrial genome, Ancient DNA, Yellow River, Neolithic

## Abstract

Shimao City is considered an important political and religious center during the Late Neolithic Longshan period of the Middle Yellow River basin. The genetic history and population dynamics among the Shimao and other ancient populations, especially the Taosi-related populations, remain unknown. Here, we sequenced 172 complete mitochondrial genomes, ranging from the Yangshao to Longshan period, from individuals related to the Shimao culture in northern Shaanxi Province and Taosi culture in southern Shanxi Province, Middle Yellow River basin. Our results show that the populations inhabiting Shimao City had close genetic connections with an earlier population in the Middle Neolithic Yangshao period of northern Shaanxi Province, revealing a mostly local origin for the Shimao Society. In addition, among the populations in other regions of the Yellow River basin, the Shimao-related populations had the closest maternal affinity with the contemporaneous Taosi populations from the Longshan period. The Shimao-related populations also shared more affinity with present-day northern Han populations than with the minorities and southern Han in China. Our study provides a new perspective on the genetic origins and structure of the Shimao people and the population dynamics in the Middle Yellow River basin during the Neolithic period.

## Introduction

Northern China is a large geographic region that encompasses the Yellow River (YR) basin, in which the residing Neolithic cultures (such as the Yangshao and Longshan cultures) laid an important foundation for the origin of Chinese civilization (Wang, 1989; Dong et al., 2016; Zhou, 2017). The Middle Neolithic (MN) Yangshao period (∼7,000–5,000 years before present, BP) was a stage of rapid development and expansion, giving rise to the Majiayao culture (∼5,700 BP) in the Upper YR (Yang, 2016), the Dahecun culture in the Middle YR (∼5,700 BP) (Xu, 2004), and the Beixin (∼5,400 BP) and Dawenkou cultures (∼6,000 BP) in the Lower YR (Zhang, 1996; Wang, 2005; Zhang, 2015; Dong et al., 2016; Duan, 2019). This cultural development and expansion coincided with the Holocene Climatic Optimum period in northern China (Hou et al., 2019). In the Late Neolithic (LN) Longshan period (∼4,500–3,800 BP), the cultural features in different regions (∼4,300 BP in Shaanxi Province; ∼4,400 BP in Henan Province; ∼4,500 BP in Shanxi Province) of the YR basin varied spatially, increased in social complexity, and formed distinct settlements with different levels of social hierarchy (He, 2004; Chang, 2005; Sun, 2016). The influences of different archaeological cultures on various regions of the YR basin changed dynamically over time, which might have been accompanied by population flow and interaction (Sun et al., 2020a).

**FIGURE 1:**
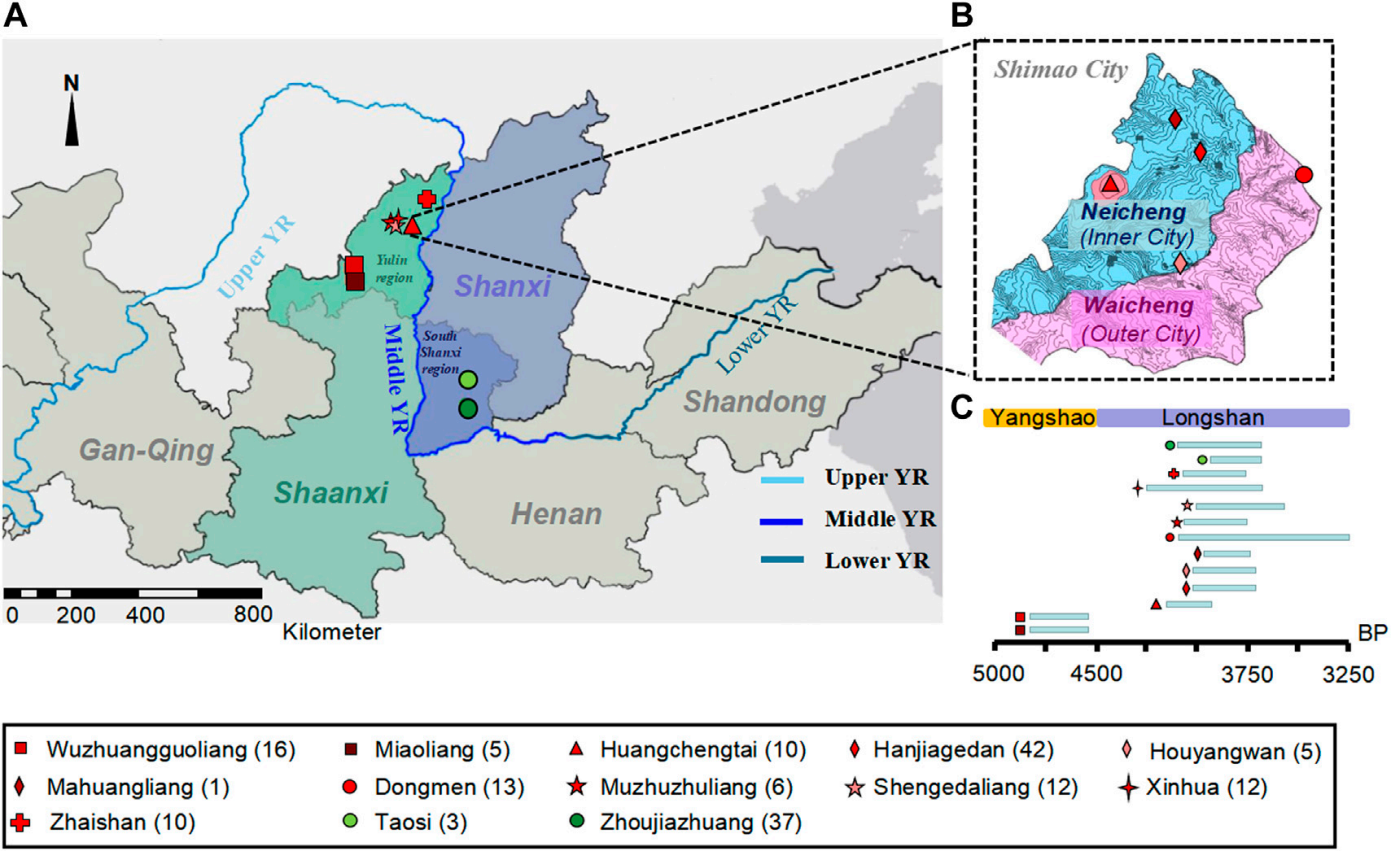
The geographical and temporal distributions of the new samples. **(A)** The geographical location of ancient individuals from 13 archaeological sites in northern Shaanxi and southern Shanxi Province of Middle YR. The different colored and shaped labels represent different sites. The dark red represents those from the MN Yangshao sites, and red represents Shimao-related populations in and neighboring Shimao City in LN Longshan period in northern Shaanxi Province; green represents the Taosi-related populations in southern Shanxi Province. **(B)** The geographical distribution of ancient individuals in Shimao City. **(C)** Timeline of archaeological sites.

The Shimao site (∼4,300–3,800 BP), also called “Shimao City”, is considered an important political and religious center during the Middle YR’s Longshan period (∼4,500–3,800 BP) (Sun et al., 2013; Rawson, 2017; Sun et al., 2020a). It is currently the largest Neolithic settlement known in China, covering 4 km^2^ with a triple structure made of stone-reinforced walls (Figure 1), and was selected as one of the world’s top 10 archaeological discoveries in the past decade (Archaeology Institute of America, 2021). The center of Shimao City, Huangchengtai, has many high-grade buildings and relics (Sun et al., 2020a; Sun et al., 2020b). The Neicheng (or “inner city”) surrounds Huangchengtai and consists of multiple grave sites (e.g., Hanjiagedan, Houyangwan, and Mahuangliang). The Dongmen (or “East Gate”) is located along the northeastern wall of Waicheng (or “outer city”) and exhibits complex fortifications (Sun et al., 2020a). According to archaeological records, these distinct sites within Shimao City showed clear differences in social hierarchy and inequality. For example, Hanjiagedan (Sun et al., 2016), Houyangwan (Sun and Shao, 2015), and those closer to Huangchengtai, yielded more high-status graves than Dongmen (Sun and Shao, 2015). Archaeologists named the “Shimao culture” based on artifacts unearthed in Shimao City (Dai, 1998; Sun et al., 2020b). The sites neighboring Shimao City in northern Shaanxi Province, such as the Muzhuzhuliang (Wang et al., 2015), Shengedaliang (Guo et al., 2016), Xinhua (Xing et al., 2002), and Zhaishan sites (Shao et al., 2021), were all attributed to the Shimao culture. However, the origin of Shimao City was still uncertain. The Shimao culture was considered to have developed from local populations with an influence from surrounding cultures; however, it may have alternatively originated from the migration of populations from the Central Plain or other regions (Dai, 1998; Zhang, 2004; Sun, 2005). In addition, recent studies revealed that the Shimao culture interacted frequently with other regions in the YR basin outside northern Shaanxi Province during the Neolithic period (Guo, 2013; Chen et al., 2016; Chen et al., 2017; Sun et al., 2020b), especially the Taosi culture in southern Shanxi Province of the Middle YR (Yan and He, 2005; Tian and Dai, 2018; Shao, 2020). The links between these two cultures may have been political, economic, cultural, or through shared population connections (Shao, 2020). However, the interactions between the Shimao and Taosi people remain ambiguous from the perspective of archaeology and physical anthropology (Yan and He, 2005; Guo, 2013; Chen et al., 2016; Chen et al., 2017; Tian and Dai, 2018; Sun et al., 2020b; Shao, 2020). Although there were some genomic analyses that included samples from the Shengedaliang (Ning et al., 2020) and Wuzhuangguoliang sites in northern Shaanxi Province (Zhao et al., 2017; Wang et al., 2021), the large-scale genetic affinities among the populations related to the Shimao culture and their predecessors, along with other populations in different regions of the YR basin, is still unclear.

In the current study, we sequenced the complete mitochondrial genomes of 172 samples from various archaeological sites, particularly the individuals related to Shimao and Taosi cultures in the Middle YR. Our research presents large-scale mitogenomic data and new perspectives for exploring the maternal genetic history and dynamics of Shimao-related populations and the populations in the Middle YR basin during the Neolithic period.

## Materials and Methods

### Ancient DNA Extraction and Library Preparation

We collected samples from a total of 172 ancient human individuals from 13 sites. We describe their archaeological details in the Supplementary Materials (Supplementary Table S1). A total of 172 DNA extractions were obtained from less than 100 mg of bones or dental remains of ancient samples. All ancient DNA work was conducted in the aDNA clean room at the Institute of Vertebrate Paleontology and Paleoanthropology, following strict aDNA standards (Gilbert et al., 2005).

We prepared single-stranded (denoted as “SS”) and double-stranded (denoted as “DS” in Supplementary Table S1) libraries and partially treated them with uracil-DNA glycosylase (“UDG”) to remove deaminated cytosine (Kircher et al., 2021; Meyer et al., 2012). Libraries were amplified for 35 cycles using AccuPrime Pfx DNA polymerase under conditions described in a previous study (Dabney and Meyer, 2012). P5 and P7 adapters were added to limit the contamination rate (Kircher et al., 2012). And the NanoDrop2000 spectrometer was used for monitoring the DNA concentration.

### Ancient DNA Capture and Sequencing

To enrich endogenous ancient DNA from the high levels of background environmental DNA, we used a DNA capture technique (Fu et al., 2013; Fu et al., 2015; Haak et al., 2015). The in-solution capture of the mitochondrial DNA (mtDNA) was accomplished by overlapping probes with DNA fragments and enriching the resulting libraries (Fu et al., 2013). The probes were synthesized based on the human mitochondrial genome.

After enrichment, the Illumina Miseq platform was used to generate 2 × 76 bp paired-end reads. The leeHom software (https://github.com/grenaud/leeHom) was used to trim adapters and merge sequences, with paired-end reads overlapping by at least 11 bp (Gabriel et al., 2014). Sequenced and merged reads with lengths of at least 30 bp were then mapped to the revised Cambridge Reference Sequence version 17 (rCRS, Genbank accession number NC_012920) for mtDNA, using the same command in the BWA v0.6.1 aligner (arguments used: -n 0.01 and −l16500) (Li and Durbin, 2009; Renaud et al., 2014). We removed duplicate sequences and retained the one with highest mapping quality. After removing sequences with a mapping quality below 30, we constructed the whole mitochondrial sequence (Supplementary Table S1).

### Test for Contamination

We evaluated the contamination rate using the ContamMix software and compared the mtDNA fragments with the consensus mitochondrial genome for our newly sampled individuals and 311 present-day world-wide sequences (Fu et al., 2013). We treated the libraries as contaminated if over 4% of the fragments matched with other sequences that are better than the consensus (Fu et al., 2016). For the libraries with substantial contamination (contamination rate > 4%), we excluded them (Briggs et al., 2007; Rohland et al., 2015). Of the 172 new mtDNA samples, 166 of them have lower contamination rates (< 4%, average 0.95%) (Supplementary Table S1).

### Kinship Analysis

We treated the mtDNA sequences as kinship-related individuals if they shared identical mitochondrial genomic sequences and were from the same tombs. The Bioedit software (version 7.2.5) was used for testing the kinship. Finally, we found four pairs of sequences with probable matrilineal kinships (Hall, 1999). We excluded the samples in each pair that had relatively lower coverage (Supplementary Table S1).

In total, we sequenced the complete mitochondrial genomes of 172 individuals. After removing those with higher contamination rates and kinships, 162 individuals sequenced to between 20.74-fold and 827.53-fold coverage (average 253.95-fold) were used for analysis (Supplementary Table S1).

### Haplogroup Analysis

MUSCLE (MUSCLE 3.8.31) and Bioedit software were used to align and edit the complete sequences of mtDNA with rCRS (Andrews et al., 1999; Edgar, 2004; Weissensteiner et al., 2016). Haplogrep2, built on Phylotree Build 17, was used to call haplogroups for each sample (van Oven, 2015; Weissensteiner et al., 2016) (Supplementary Table S1). We grouped all haplogroups absent from the populations in and around Shimao City as “Other”. Since the haplogroups R and N distribute in both East and West Eurasian, we used R^#^ and N^#^ (such as haplotype R+16189, sub-haplogroups R11 and N9, which were also observed in the Shimao-related populations) to represent the haplogroups carried by East Eurasian in our dataset. The other sub-haplogroups R and N (such as sub-haplogroups R1, R2, N1, N2) found in West Eurasians, were assigned to “Other.”

### Principal Component Analysis and Haplogroup Sharing

In addition, we calculated haplogroup frequencies for every group and conducted a PCA using the built-in function “prcomp” in R (version 4.1.2) software (Venables and Ripley, 2002). We plotted PC1 and PC2 to illustrate the haplogroup differences among populations and explore the maternal genetic relationship among the populations.

We calculated haplogroup sharing as a pairwise matrix of proportions of haplogroups shared among populations. In the matrix, the entries represent the proportions of haplogroups shared in the two populations, which are calculated by summing the shared frequencies of all the same haplogroups between them (Ko et al., 2014; Miao et al., 2021). The proportions of each population were normalized by dividing by the total count and summing to one (Ko et al., 2014).

### Discriminant Analysis of Principal Components

We also used discriminant analysis of principal components (DAPC), which maximizes the inter-variation between groups while minimizing the intra-variation, to show the maternal genetic relationships among ancient populations in the YR basin (Pritchard et al., 2000). We used the DAPC function of the “adegenet” package in R (version 4.1.2) software to conduct a sequence–based DAPC (Jombart et al., 2010).

### Genetic Distance Analysis

The Arlequin software package (version 3.5.2.2) was used to calculate genetic distances (*F*_ST_) between populations (Excoffier et al., 2007), which were visualized with the package “pheatmap” in R. Generally, a lower *F*_ST_ represents a closer maternal genetic relationship between two groups. Heatmaps were also drawn to illustrate the statistical significance of clusters based on *F*_ST_.

### Haplotype Network Construction

To explore the genetic relationship of certain haplotypes in samples, we used DNASP6 (version 6.12.03) and PopArt 1.7 to conduct a median-joining network analysis on all the samples in the same haplogroup (sub-haplogroup or haplotype) dataset (Bandelt et al., 1999; Leigh and Bryant, 2015) and construct the haplotype network graph. This helped us understand the inflow or diffusion process of a haplotype population.

## Results

### Sample and Ancient DNA Generation

We captured mitochondrial DNA (mtDNA) from 172 ancient individuals from 13 archaeological sites in the northern Shaanxi and southern Shanxi Provinces of the Middle YR (Figure 1A; Supplementary Table S1), with dates ranging from 4,836 to 3,253 calibrated BP (cal BP). Removing six individuals with high contamination rates (> 4%) and four individuals owning close relatives (defined as the same mtDNA sequences), resulted in a final dataset of 162 individuals with coverage between 20.74- and 827.53-fold (Supplementary Table S1).

Among these new samples, we obtained 21 samples from the Miaoliang and Wuzhuangguoliang sites (referred to as the “preShimao_MW” group) in northern Shaanxi Province, dating to 4,836–4,530 cal BP in the MN Yangshao period. Additionally, we obtained 91 samples from the LN Longshan period of northern Shaanxi Province, of which 66 were from Shimao City and 35 were from sites neighboring Shimao City (Supplementary Table S1). We grouped the individuals in Shimao City based on their archaeological cultures, dates, and geographical locations within Shimao: 10 individuals were excavated from the political and religious center, Huangchengtai site, which we named “Shimao_HCT” (4,148–3,895 cal BP); 44 individuals were from the Neicheng (or “inner city”), which we grouped as “Shimao_NC” (3,977–3,699 cal BP) and contained individuals from the Hanjiagedan, Houyangwan, and Mahuangliang sites; and 12 individuals from the Dongmen site in Waicheng (also called the “East Gate in outer city”), which we named “Shimao_DM” (4,144–3,253 cal BP). The individuals excavated from the Xinhua (XH, n = 9, 4,231–3,650 cal BP), Muzhuzhuliang (MZZL, n = 4, 4,082–3,722 cal BP), Shengedaliang (SGDL, n = 12, 3,969–3,570 cal BP), and Zhaishan (ZS, n = 10, ∼4,050–3,750 BP) sites neighboring Shimao City, we named as the abbreviations of their site names. We integrated MZZL and SGDL into “MZZSGDL” (n = 16) for their similar archaeological cultures, locations, and dates, and the small population size (n = 4) from the MZZL site.

In addition, we sequenced mtDNA from 40 LN individuals excavated from the “TSZJZ” group (∼4,150–3,696 BP) related to Taosi culture (containing Taosi and Zhoujiazhuang sites) in the southern Shanxi Province of the Middle YR.

We also collected 801 previously published mtDNA sequences for the ancient individuals from East and West Eurasia, ranging from the Early Neolithic (EN) to Historic Era (HE). These included populations from Xinjiang (∼5,000–500 BP), Gansu and Qinghai Provinces (∼5,040–411 BP), Henan Province (∼5,500–5,000 BP, Qingtai site), Shandong Province (∼9,600–2,000 BP), the Tibetan Plateau (∼3,000–100 BP), southern East Asia (∼4,600–300 BP), the Baikal River in southern Siberia (∼7,123–6,319 BP and ∼4,860–3,760 BP), Mongolia (∼3,330–2,950 BP and ∼2,147–2,007 BP), and the Steppe and West Eurasia (∼5,450–1,500 BP). Meanwhile, we also obtained the haplogroup information from individuals in Inner Mongolia (∼4,500 BP, Halahaigou site) (Supplementary Table S2).

For the present-day populations, we collected 7,641 individuals from northeastern Asians (NEAs, including North Asians and northern East Asians), southeastern Asians (SEAs, Southeast Asians and southern East Asians), and central-west Eurasians (CWEs). Among these populations, 2,102 individuals from China, including 388 Han individuals from northern China and 168 individuals from southern China, which we named “NChina_Han” and “SChina_Han”, respectively. We also collected 548 individuals from 16 ethnic minorities, which covers the vast majority of ethnic minorities in China (Supplementary Table S3). We served them as different groups following their minorities. The present-day populations also contained the populations in Tibet (“SChina_Tibet”) and Taiwan (“SChina_Taiwan”) (Ko et al., 2014; Kang et al., 2016).

### The Mostly Local Genetic Origin of Shimao Populations From Earlier Populations in Northern Shaanxi Province

To understand the genetic connection between the LN Shimao populations and the preceding populations in the MN period, we collected 21 individuals from the MN preShimao_MW sites and 66 individuals from the LN Shimao City in northern Shaanxi Province (Figure 1).

**FIGURE 2:**
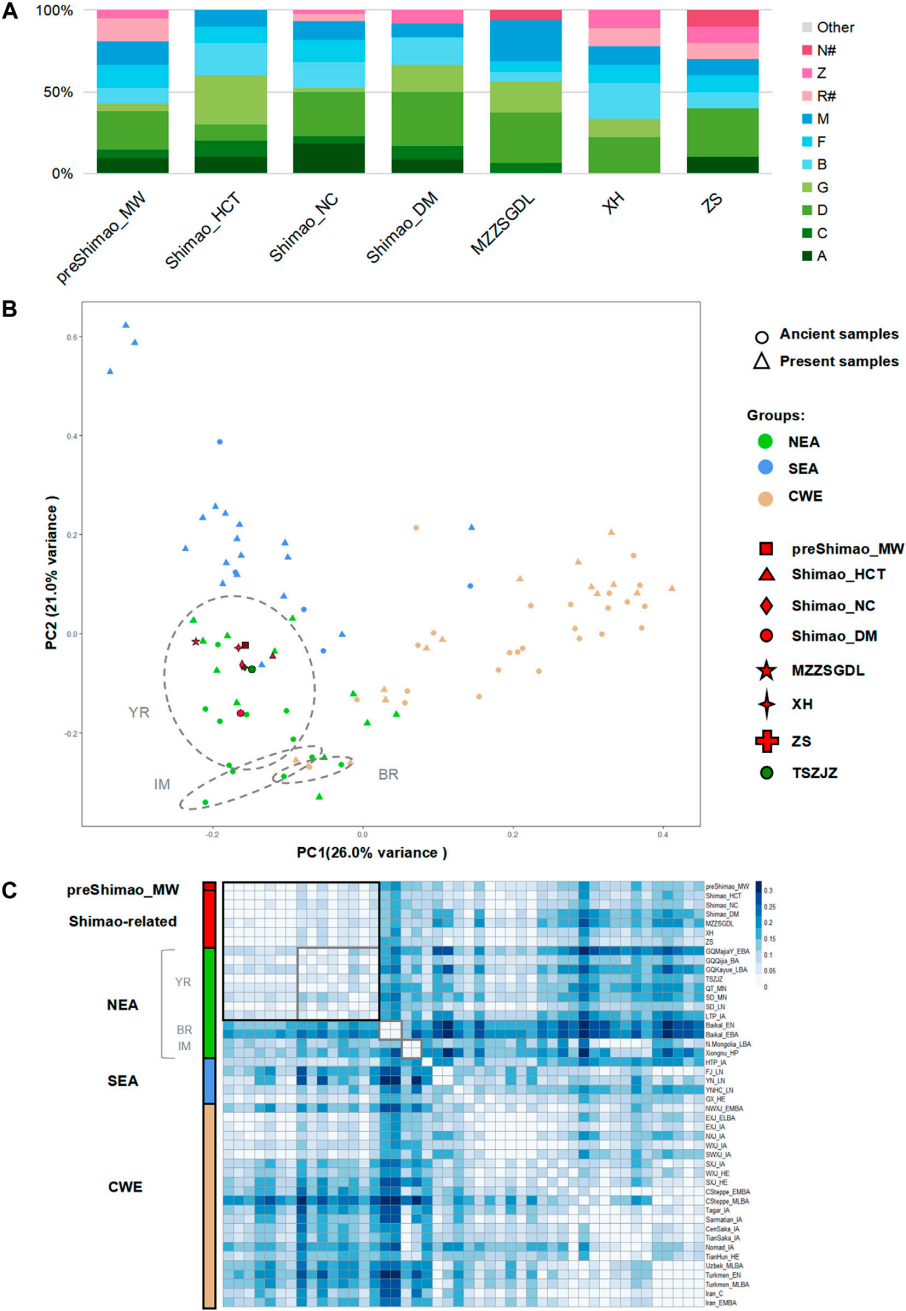
The genetic analysis of ancient populations in northern Shaanxi Province. **(A)** Haplogroup frequency. The haplogroups with green are those common in northeastern Asians (NEAs), and those with blue are common in southeastern Asians (SEAs). The haplogroups absent from Shimao-related populations are grouped in “Other.” The haplogroups R^#^ and N^#^ represent the haplotypes shown in East Eurasians (such as haplotype R+16189, sub-haplogroups R11 and N9, which were observed in the Shimao-related populations). **(B)** Principal Component Analysis (PCA) based on the haplogroup frequencies. The circle and triangle shapes represent ancient and present-day populations, respectively. The colored and shaped symbols correspond to Figure 1A. The grey circles represent the ancient populations from the Yellow River basin (YR), Mongolia and Inner Mongolia (IM), and the Baikal Lake region (BR) in NEA. CWE: Central and Western Eurasian. **(C)** The genetic distance (*F*_ST_) heatmap of Shimao-related populations and other ancient populations. The different labels and colors correspond to the PCA plot, and different shades of color are used to mark different regional populations. Values with *F*_ST_ = 0.00 are in white, representing a close genetic relationship. SD_EN were excluded in the heatmap because of the significantly large genetic distance (*F*_ST_ > 0.10) between them and other populations. The grey squares represent the ancient populations from the Yellow River basin (YR), Mongolia and Inner Mongolia (IM), and the Baikal Lake region (BR) in NEA.

**FIGURE 3:**
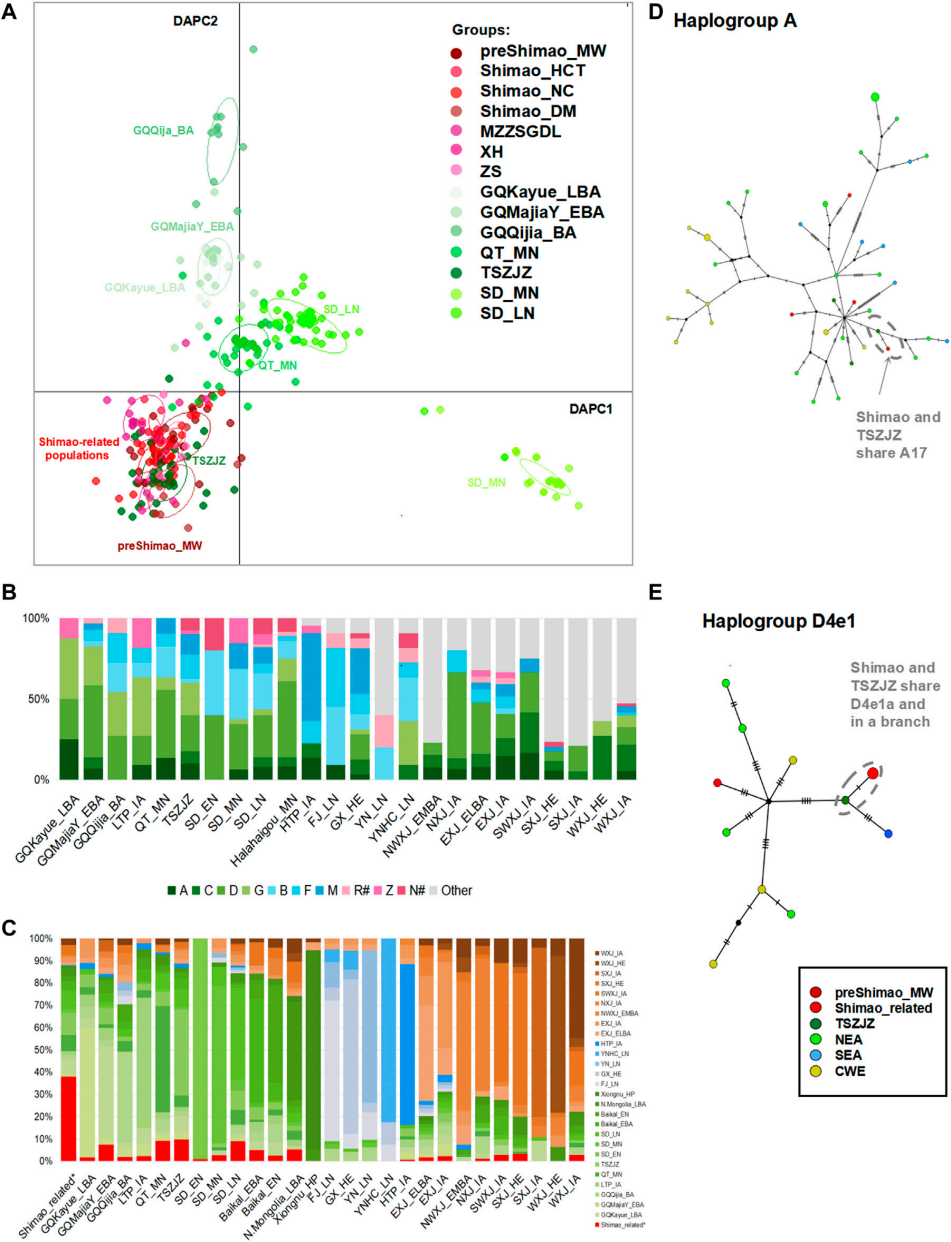
The genetic analysis between Shimao-related populations and other ancient populations. **(A)** The discriminant analysis of principal components (DAPC) of ancient populations in YR. The red points represent the Shimao-related populations. The dark green points represent the TSZJZ individuals, and the other green points represent the individuals in YR. **(B)** Haplogroup frequency of ancient populations. The haplogroups with green are those common in NEAs, and those with blue are common in SEAs. The haplogroups absent from Shimao-related populations are grouped in “Other.” The haplogroups R^#^ and N^#^ represent the haplotypes found in East Eurasians (such as haplotype R+16189, sub-haplogroups R11 and N9, which were also observed in the Shimao-related populations). The other sub-haplogroups R and N found in West Eurasians were assigned to “Other.” **(C)** Haplogroup sharing analysis. The different colors correspond to *F*_ST_ heatmap and networks. Median-joining networks of haplotypes A17 **(D)** and D4e1a **(E)** related to ancient northern Chinese populations. The different population groups are shown in different colors that are consistent with those groups in the *F*_ST_ heatmap.

The haplogroup analysis found that ancient and present-day NEAs, showed a high proportion of haplogroups A (maximum, 71.43%), C (maximum, 55.00%), D (maximum, 60.00%), and G (maximum, 37.50%) with a north-south declining trend (Supplementary Figure S1; Supplementary Tables S4, S5). Haplogroups B (maximum, 36.36%, B4′5), F (maximum, 40.00%), and M (maximum, 83.33%) were common in ancient and present-day SEA and showed a north-south increasing trend (Supplementary Figure S1; Supplementary Tables S4, S5). The earlier population in the MN period of northern Shaanxi Province (4,836–4,530 cal BP), preShimao_MW, carried the haplogroups A (9.52%), C (4.76%), D (23.81%), G (4.76%), B (9.52%, B4′5), F (14.29%), M (14.29%), Z (4.76%), and R^#^ (14.29%), and showed a higher proportion of NEA (rather than SEA) haplogroups (Figure 2A). Our Principal Component Analysis (PCA) based on haplogroup frequency showed that the PC1 explains population variation from east to west geographically and that PC2 explains the variation from north to south (Figure 2B). In general, all the populations are genetically divided into three clusters: NEA, SEA, and CWEs. The preShimao_MW was distributed among the NEA populations and clustered with the populations in the YR (Figure 2B). In addition, this MN population showed the highest proportion of haplogroup D (23.81%), which was also found in relatively higher proportions in the YR populations (18.18–44.83%) (Figure 2A, Supplementary Table S4). The *F*_ST_ heatmap based on genetic distance also showed that the preShimao_MW clustered with the YR populations (Figure 2C). Thus, the MN Yangshao populations from northern Shaanxi Province (preShimao_MW) were more related to the NEA populations in the YR basin than to populations from other regions in East Asia. Although there were no significant genetic affinities between the preShimao_MW and the EN and MN YR populations (*F*_ST_ > 0.06, *p* < 0.01 with QT_MN and SD_MN; and *F*_ST_ = 0.31, *p* > 0.07 with SD_EN), the DAPC shows some overlap between the preShimao_MW and the QT_MN from the middle YR (Figure 3A; Supplementary Table S6). The same haplotypes G3a2, D5a2a1, and F1a1c were also observed in both preShimao_MW and QT_MN, suggesting some connections between them (Supplementary Figure S2; Supplementary Tables S1, S2).

For the LN Longshan populations (4,148–3,253 cal BP) in Shimao City, including Shimao_HCT, Shimiao_NC, and Shimao_DM, the haplogroup analysis showed that they carried similar haplogroups: A (8.33–18.18%), C (4.55–10.00%), D (10.00–33.33%), G (2.27–30.00%), B (15.91–20.00%, B4′5), and M (8.33–11.36%) (Supplementary Table S4). The Shimao populations also displayed higher proportions of NEA dominating haplogroups than SEA dominating haplogroups, and Shimao_DM (66.67%) showed a higher ratio of NEA dominating haplogroups than Shimao_HCT (60.00%) and Shimao_DM (52.27%). Moreover, Shimao_NC additionally carried haplogroup R^#^ (4.55%). Shimao_NC and Shimao_DM also had the highest proportions of haplogroup D (27.27–33.33%), similar to preShimao_MW and most YR populations (Figure 2A; Supplementary Table S4). The PCA shows that the three Shimao populations clustered and plot among the NEA populations in the YR region, consistent with the haplogroup analysis results (Figures 2A,B). Also, we found that the genetic distances (*F*_ST_ values) among the three Shimao populations were all about zero (*F*_ST_ < 0.01, *p* > 0.05), revealing a close genetic affinity among them (Figure 2C; Supplementary Table S6). The same haplotypes (B4a4, C4a2, G2a1, and G1c) were found in these three populations, further suggesting a close relationship among them (Supplementary Figure S2). These results all suggest that the populations in different regions of the LN Shimao City shared close affinities with each other.

We also explored the genetic connections between the LN Longshan populations (4,148–3,253 cal BP) in Shimao City and the earlier populations (before 4,500 BP) in and outside of northern Shaanxi Province. We found that the MN preShimao_MW and LN Shimao populations in northern Shaanxi carried similar haplogroups (A, C, D, G, Z, B, F, and M) and that Shimao_HCT and Shimao_DM both have the highest proportions of haplogroup D, in common with preShimao_MW (Figure 2A). Some of those haplogroups were absent from the early populations outside Shaanxi Province. For example, the QT_MN (∼5,500–5,000 BP), SD_EN (∼9,600–7,700 BP), and SD_MN (∼5,500–4,600 BP) lacked haplogroup C, and SD_EN and SD_MN lacked haplogroup F (Figure 3B; Supplementary Table S4). The DAPC also indicates that the Shimao populations clustered with the preceding population (preShimao_MW) in (but not outside) northern Shaanxi Province (Figure 3A). Moreover, the three Shimao populations clustered with the preceding MN populations (preShimao_MW) in the *F*_ST_ heatmap and showed the smallest genetic differentiation between them (*F*_ST_ < 0.01, *p* > 0.05), while showing larger *F*_ST_ values with the early populations (QT_MN, SD_EN, and SD_MN) outside northern Shaanxi (*F*_ST_ > 0.05) (Supplementary Table S6). These results indicate that the LN Longshan populations in Shimao City showed the closest genetic affinity with the earlier MN Yangshao populations (preShimao_MW, 4,836–4,530 cal BP) in (but not outside) northern Shaanxi Province. This close relationship was also demonstrated by the shared haplotypes between preShimao_MW and Shimao-related populations, including D4j3 and D4b2b of D4 and haplotypes A+152 + 16362, F1a1c, and R11, and by plotting on the same branches of the median-joining network (Supplementary Figure S2). However, we also found some connections between the LN Shimao populations and the earlier QT_MN from the middle YR. This is supported by the appearance of haplotypes M9a1a1 and M10a1b, which differed in these two populations by only one and four mutations, respectively, in the network analysis (Supplementary Figure S2).

Therefore, the populations in different regions of the Longshan period’s Shimao City (4,148–3,253 cal BP) shared close affinities with each other and with the preceding MN Yangshao populations (4,836–4,530 cal BP) in (rather than outside) northern Shaanxi Province. The results reveal that the MN Yangshao populations in northern Shaanxi Province were largely not replaced with the foundation of Shimao City, supporting a hypothesis of a mostly local genetic origin for the Shimao people. However, given the shared haplotypes with other YR populations (i.e., the MN Qingtai), we cannot rule out additional genetic contribution from populations outside northern Shaanxi province.

### The Genetic Affinities Among Populations in and Around Shimao City in Northern Shaanxi

The archaeological reports indicated that the LN Longshan sites around Shimao City in northern Shaanxi Province, such as the MZZSGDL, XH, and ZS, were all related to the Shimao culture. To explore the genetic affinities among the contemporaneous LN populations related to Shimao culture (containing the populations in and around Shimao City), we sequenced 35 new samples from the sites neighboring Shimao City.

These three populations (MZZSGDL, XH, and ZS) around Shimao City primarily carried haplogroups D (22.22–31.25%), B (6.25–22.22%, B4′5), F (6.25–11.11%), and M (10.00–25.00%). Moreover, MZZSGDL also carried haplogroups C (6.25%) and G (18.75%), XH carried haplogroups G (11.11%), Z (11.11%), and R^#^ (11.11%), and ZS had haplogroups A (10.00%) and R^#^ (10.00%). All three populations displayed the highest proportions of haplogroup D (31.25% in MZZSGDL, 22.22% in XH, and 30.00% in ZS) (Figure 2A; Supplementary Table S4). In the PCA plot, these populations neighboring Shimao City plot among the NEA populations in YR region and closer to each other (Figure 2B). This close relationship was also revealed by the smaller genetic differentiation between them (*F*_ST_ < 0.01) (Figure 2C; Supplementary Table S6). In the median-joining networks, these populations shared the same branches in haplotype Z3, F2g, and M10a1a1b (Supplementary Figure S2). Thus, these three populations neighboring Shimao City were closely related.

In addition, we found that these populations neighboring Shimao City showed the same haplogroups (haplogroups D, B (B4′5), F, and M) as the populations in Shimao City, and some of them share haplotypes (A+152 + 16362, B4a4, D4j3, F2g, and Z3) of the same branch (Figures 2A,D; Supplementary Figure S2). We also found this close affinity in the DAPC and *F*_ST_ heatmap, as well as through the smaller genetic distance between them (*F*_ST_ = 0.00, *p* > 0.05 in most of them, and *F*_ST_ = 0.04, *p* < 0.05 between Shimao_NC and MZZSGDL) (Figures 2C, 3A; Supplementary Table S6). Among the earlier populations (before 4,500 BP), the individuals neighboring Shimao City showed the closest relationship with the MN Yangshao populations (preShimao_MW) in (rather than outside) northern Shaanxi Province, similar to the populations in Shimao City, indicated by their shared haplogroups, close distributions in the DAPC, and the smaller *F*_ST_ values between them (*F*_ST_ = 0.00, *p* > 0.05 between preShimao_MW and XH, ZS; and *F*_ST_ = 0.04, *p* = 0.03 between preShimao_MW and MZZSGDL) (Figures 2A,C, 3A; Supplementary Table S6). Similar with Shimao populations, the populations neighboring Shimao City (such as, MZZSGDL) indicated some slight connections with MN Qingtai supported by the same haplotype M9a1b, which only showed four-mutation differences between them in the network analysis (Supplementary Figure S2). Thus, these results show that the populations in and around Shimao City shared close genetic affinities.

Above all, during the LN Longshan period, the populations related to Shimao culture (containing the populations in and around Shimao City, which we call “Shimao-related populations”) showed close affinities with each other, revealing the extensive connections among the populations not only in but also around Shimao City in northern Shaanxi Province. All Shimao-related populations were shown to have a mostly local genetic origin from the preceding MN Yangshao populations in northern Shaanxi Province.

### The Maternal Affinity Between the Populations Related to Shimao and the Contemporaneous Taosi Culture in the Middle Yellow River

Given the close genetic relationship between the MN Yangshao (4,836–4,530 cal BP) and LN Longshan period (4,231–3,253 cal BP) in northern Shaanxi Province, we then focused on the population interactions between the LN Shimao-related populations and the ancient humans outside northern Shaanxi Province. Previous archaeological studies had shown that stone carvings excavated from Shimao City shared cultural characteristics with the Shang Dynasty (3,500–2,900 BP) in the Central Plain of the Middle YR. Meanwhile, research on pottery from Shimao City found that the Shimao culture was closely associated with the contemporaneous Taosi culture in the southern Shanxi Province of the Middle YR (Shao, 2020; Sun and Shao, 2020). To explore the genetic relationship between Shimao-related populations and the populations in different regions of the YR, we sequenced 40 new individuals from TSZJZ related to the Taosi culture in the southern Shanxi Province of the Middle YR and collected 198 previously published ancient individuals from different regions of the YR.

We found that the Shimao-related populations shared more affinities with those NEA populations in the YR basin. Among those YR populations, the early Bronze Age (EBA) individuals in the Upper YR (GQMajiaY_EBA) and the LN individuals in the Middle (TSZJZ) and Lower YR (SD_LN), which date in and after the LN Longshan period (after 4,500 BP), carried higher proportions of haplogroups common in NEAs, such as haplogroups A (6.90–10.00%), C (6.00–7.50%), D (22.50–44.83%), and G (4.00–24.14%). Among these haplogroups, haplogroup D showed the highest proportion in these three YR populations (22.50% in TSZJZ, 26.00% in SD_LN, and 44.83% in GQMajiaY_EBA). In addition, they all have the haplogroups B (2.50–22.00%, B4′5), F (6.00–15.00%), and M (3.45–12.50%) (Figure 3B; Supplementary Table S4). These haplogroups were all found in the Shimao-related populations (Figures 2A, 3B). In the DAPC plot, among the Middle YR populations, the Shimao-related populations were closer to the contemporaneous LN Longshan populations (after 4,500 BP) related to the Taosi culture (TSZJZ, 4,150–3,696 cal BP) than to the MN Yangshao populations (∼5,500–5,000 BP) from the Qingtai site (QT_MN, ∼5,500–5,000 BP) (Figure 3A). Among the Lower YR populations, the Shimao-related populations were closer to the LN Longshan populations (SD_LN, after 4,500 BP) than to the EN (SD_EN, ∼9,600–7,700 BP) and MN (SD_MN, ∼5,500–4,600 BP) individuals (Figure 3A). The DAPC suggests that the Shimao-related populations were closer to the contemporaneous LN (after 4,500 BP), rather than the EN and MN (before 4,500 BP) populations outside northern Shaanxi Province in the YR basin. The *F*_ST_ results also showed smaller genetic differentiation between Shimao-related individuals and the contemporaneous LN Longshan populations (*F*_ST_ = 0.00, *p* > 0.05 with TSZJZ; *F*_ST_ < 0.02, *p* > 0.02 between most Shimao-related populations and SD_LN; *F*_ST_ = 0.05, *p* = 0.00 between MZZSGDL and SD_LN) than with those earlier populations in the YR region (*F*_ST_ = 0.04–0.07, *p* < 0.05 with QT_MN; *F*_ST_ = 0.04–0.80, *p* < 0.05 with SD_MN; and *F*_ST_ = 0.12–0.49, *p* > 0.05 with SD_EN) (Supplementary Table S6). The haplogroup sharing analysis also indicated that the YR populations during the LN shared higher proportions of haplotypes with Shimao-related populations than those in EN and MN (9.80% in TSZJZ, whereas 9.27% in QT_MN of Middle YR region; 9.05% in SD_LN, whereas 0.98% in SD_EN and 2.79% in SD_MN of Lower YR region) (Figure 3C; Supplementary Table S7). These results also suggest that the Shimao-related populations were closer to LN populations (after 4,500 BP) than the earlier populations (before 4,500 BP) found elsewhere in the YR.

Among the LN populations across the YR, the populations related to the Shimao culture were closest to those related to the Taosi culture (*F*_ST_ = 0.01, *p* > 0.05 in TSZJZ) (Supplementary Table S6). Similarly, the DAPC results showed Shimao-related populations clearly clustered with TSZJZ to the exclusion of other LN and BA populations (Figure 3A). In addition, the haplogroup sharing analysis also indicated that the Taosi-related individuals shared slightly higher proportions (9.80% in TSZJZ; 9.05% in SD_LN) of haplotypes with the Shimao-related populations (Figure 3C; Supplementary Table S7). The network results further showed that the haplotypes carried by TSZJZ, such as A17, C4a1a2, C4a2a1, D4b2b, D4e1a, F1a1c, and F2g, were also found in Shimao-related populations and that they shared the same branches (Figures 3D,E; Supplementary Figure S2). These results indicate that, among the populations in other regions of the YR basin, the Shimao-related populations had the closest genetic affinity with the Taosi culture-related populations in southern Shanxi Province.

In all, the ancient individuals related to the Shimao culture in the LN Longshan period from northern Shaanxi Province shared more maternal relationships with the contemporaneous (but not earlier) populations in the YR region outside northern Shaanxi Province. Among these LN Longshan populations, those related to Shimao culture shared the closest relationship with those related to Taosi culture in the Middle YR. These results demonstrate the strong and extensive population interactions during the LN Longshan period, not only within the northern Shaanxi Province but also between northern Shaanxi and southern Shanxi Provinces.

### The Genetic Relationship Between Shimao-Related Populations and Present-Day Humans

To explore the genetic relationships between Shimao-related populations and present-day humans, we compared their genetic affinities including the ethnic minorities (such as Daur, Mongolia, Dai, Miao, etc.), Han populations (“NChina_Han” and “SChina_Han”), and the populations in Tibet and Taiwan of China.

**FIGURE 4:**
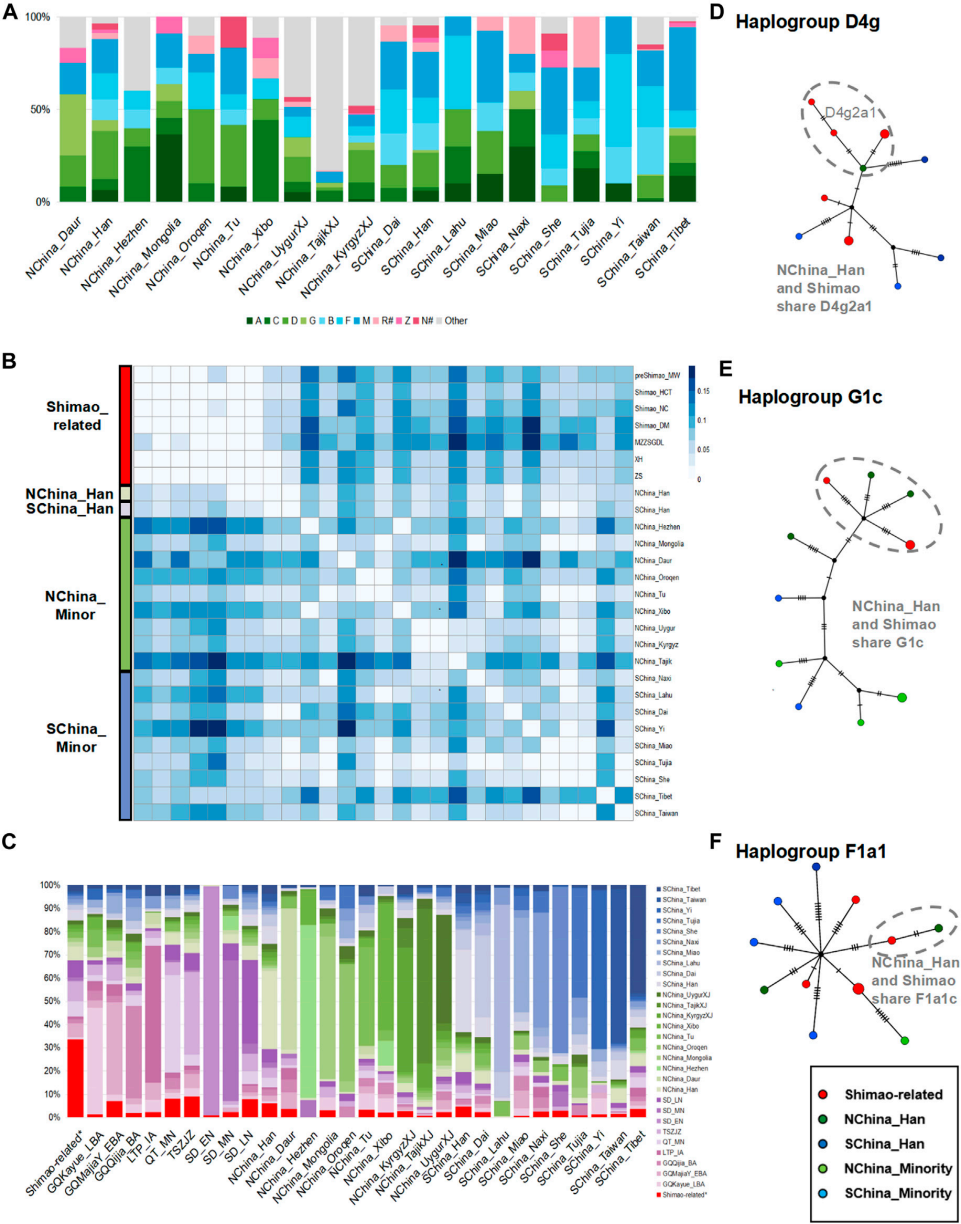
The genetic analysis between Shimao-related populations and present-day Chinese populations. **(A)** Haplogroup frequencies. The haplogroups with green are those common in NEAs, and those with blue are common in SEAs. The haplogroups absent from Shimao-related populations are grouped in “Other.” The haplogroups R^#^ and N^#^ represent the haplotypes found in East Eurasians (such as haplotype R+16189, sub-haplogroups R11 and N9, which were also observed in the Shimao-related populations). The other sub-haplogroups R and N found in West Eurasians were assigned to “Other.” **(B)** Genetic distance (*F*_ST_) heatmap of Shimao-related populations and present-day populations. NChina_Minor: the minorities in northern China; SChina_Minor: the minorities in southern China, SChina_Tibet: the populations in Tibet; SChina_Taiwan: the populations in Taiwan. **(C)** Haplogroup sharing analysis. Green and blue are used to represent populations in northern and southern China. Purple represents the ancient populations in YR. Median-joining networks of haplotypes D4g2a1 **(D)**, G1c **(E)**, and F1a1c **(F)** shared between Shimao-related populations and northern Han populations. Different colors of groups correspond to the heatmap in Figure 4B.

Among these present-day populations in China, the Han populations carried both the NEA dominating haplogroups A (5.95–6.46%), C (1.79–5.94%), D (19.05–25.84%), and G (1.19–5.94%) and SEA dominating haplogroups B (11.37–14.88%, B4′5), F (13.69–13.95%), and M (18.60–24.40%), and showed the highest proportion of haplogroup D (19.05%–25.84%), consistent with the Shimao-related populations (Figure 4A; Supplementary Table S5). The genetic distance analysis also showed that the Shimao-related populations (such as, Shimao_HCT, XH, ZS) were closer to Han populations (*F*_ST_ < 0.03, *p* > 0.06 in NChina_Han; *F*_ST_ < 0.04, *p* > 0.06 in SChina_Han) than other present-day minority populations, including those in Tibet and Taiwan (Figure 4B; Supplementary Table S6). The haplogroup sharing analysis showed that the Shimao-related populations shared higher proportions of haplotypes with the Han (NChina_Han, 6.04%; SChina_Han, 4.70%) than with the other present-day populations (0.00–3.65%) (Figure 4C; Supplementary Table S8). Additionally, the Shimao-related populations (such as, Shimao_HCT, XH, ZS) were genetically closer to northern Han populations (NChina_Han, *F*_ST_ < 0.03, *p* > 0.06) than the southern Han populations (SChina_Han, *F*_ST_ > 0.03, *p* > 0.03) (Figure 4B; Supplementary Table S6). Moreover, the haplogroup sharing analysis also demonstrated that the northern Han population shared higher proportions (NChina_Han, 6.04%) of haplotypes with Shimao-related populations than southern Han populations (SChina_Han, 4.70%) (Figure 4C; Supplementary Table S8). The network analysis also indicated that the Shimao-related populations and northern Han populations shared multiple haplotypes (D4g2a1, G1c, and F1a1) between them (Figures 4D–F). Thus, we conclude that the Shimao-related populations were closer to Han populations in northern China than to the minorities and Southern Han populations in China.

To explore which ancient populations had the closest genetic to the northern Han in China, we compared the affinities of our new Shimao-related populations and the ancient individuals in other regions of China to the northern Han. These include the EBA (GQMajiaY_EBA), BA (GQQijia_BA), late BA (GQKayue_LBA), and IA (LTP_IA) individuals from Gansu-Qinghai Province; the MN individuals (QT_MN) from Henan Province; the LN (TSZJZ) individuals from southern Shanxi Province; and the EN, MN, and LN individuals in Shandong Province (SD_EN, SD_MN, SD_LN). We found that the haplogroups A, C, D, G, Z, B (B4′5), F, M, and R^#^ were observed in the Shimao-related populations, LN Shandong individuals, and NChina_Han, whereas some of those haplogroups were absent from the other populations (Figures 2A, 3B, 4A). For example, TSZJZ lacked haplogroup R^#^, SD_MN lacked haplogroups C, F, and R^#^, and QT_MN lacked haplogroups C, R^#^, and Z (Figure 3B; Supplementary Table S4). Moreover, most of the Shimao-related populations (22.20–33.30%), SD_LN (26.00%), and NChina_Han (25.84%) carried the highest proportions of haplogroup D (Supplementary Tables S4, S5). The haplogroup sharing analysis showed that NChina_Han shared the highest proportions of haplotypes with Shimao-related populations (6.04%) compared to QT_MN (4.09%), TSZJZ (4.18%), and SD_LN (5.75%) (Figure 4C; Supplementary Table S8). The genetic distance analysis also showed that NChina_Han shared the closest genetic affinities with the Shimao-related populations (*F*_ST_ = 0.02, *p* = 0.10 in XH and ZS; *F*_ST_ = 0.03, *p* = 0.06 in Shimao_HCT) compared to QT_MN (*F*_ST_ = 0.03, *p* = 0.00); TSZJZ (*F*_ST_ = 0.05, *p* = 0.00); SD_LN (*F*_ST_ = 0.06, *p* = 0.00) (Supplementary Table S6). Thus, the Han populations in northern China shared more affinity with the Shimao-related populations than with the other published ancient individuals in China.

We found that the populations in and around Shimao City were closer to the northern Han Chinese populations than to the southern Han Chinese and minority populations. Compared to the other ancient individuals in China, we also found that these Han populations in northern China were closer to the Shimao-related populations.

## Discussion

Archeological research on Shimao City and the corresponding cultures reveal its importance, particularly in the Longshan period (∼4,500–3,800 BP), as a crucially political and religious center in northern Shaanxi Province of the Middle YR (Sun et al., 2020a). However, the genetic origins of the Shimao people rested on the relationships between the populations related to the Shimao culture and the local populations in the preceding MN Yangshao period, along with the other ancient populations (especially those contemporaneous) in the YR basin outside northern Shaanxi Province. In the current study, we presented a large-scale dataset of ancient mitochondrial genomes from the ancient populations in northern Shaanxi and southern Shanxi Province of the Middle YR, especially those related to the Shimao and contemporaneous Taosi cultures. Through our new study, we have characterized the genetic structure and population dynamics of the Shimao-related populations and how populations changed from the MN Yangshao to the LN Longshan period through the present day.

First, previous genomic research on the populations in the Henan Province of the Middle YR found distinct genetic composition changes from the MN Yangshao period (∼5,550–5,050 BP) to the LN Longshan period (∼4,275–3,844 BP), with the latter having more ancestry from the south (Ning et al., 2020). Similarly, the ancient populations in the Shandong region of the Lower YR also exhibited changes in their mitochondrial genomes from the Yangshao (∼5,500–4,600 BP) to Longshan (after 4,500 BP) period (Liu et al., 2021). In contrast, our results indicate that the populations in the LN Longshan period related to the Shimao culture (4,231–3,253 cal BP) in northern Shaanxi Province of Middle YR showed the closest genetic connections with their earlier populations in the MN Yangshao period (Wuzhuangguoliang and Miaoliang sites, 4,836–4,530 cal BP). This is consistent with archaeological studies showing that the relics excavated in the Longshan period maintained the features of those in the Yangshao culture in this region, differentiating them from the Central Plain (Xing et al., 2002).

The origin of Shimao City was also uncertain (Sun et al., 2020a). Some researchers believed it was developed by the local populations and influenced by surrounding archaeological cultures (Gong, 2018), while some hypothesized that populations migrated from the southern region of the Middle YR and built Shimao City (Zhang, 2004; Duan and Dong, 2018). Interestingly, the close genetic connections between Shimao populations in the LN Longshan period and the preceding populations in the MN Yangshao period (Wuzhuangguoliang and Miaoliang sites, 4,836–4,530 cal BP) in our study support a mostly local origin for Shimao and being primarily developed by the Yangshao populations before 4,500 BP in northern Shaanxi Province. However, we also find evidence for some connections between the populations in (MN preShimao_MW and LN Shimao) and outside (Qingtai in Central Plain) of northern Shaanxi Province. Thus, despite the close connection between Shimao and its MN predecessors in northern Shaanxi Province, we cannot exclude the possibility of additional genetic contribution from the middle YR region. Future studies will clarify these relationships.

Second, the archaeological studies on potteries, tiles, and other relics unearthed from Shimao City revealed the populations in Huangchengtai was the core palaces with high hierarchy, while the populations in the Dongmen of Waicheng were mainly from sacrificial pits, which were the sacrificed victims with low social status (Sun and Shao, 2020). However, our findings revealed that these populations in different regions of Shimao City showed close genetic affinities, though they had different levels of inferred social status. Those populations in Xinhua, Muzhuzhuliang, Shengedaliang, and Zhaishan sites related to Shimao culture had varying levels of social hierarchy and also showed a close relationship with the ancestors of the Shimao people, indicating the extensive connections among the Shimao-related populations. Thus, although the LN Longshan populations in and around Shimao showed varying levels of social hierarchy, they also shared close genetic connections with each other, consistent with their proposed cultural connections (Sun et al., 2020a).

Moreover, the physical anthropology of the individuals in Dongmen suggested that their skulls were similar to those related to the Lower Xiajiadian culture (∼4,150–3,590 BP) in Inner Mongolia (Chen et al., 2016). Published mitochondrial haplogroups from individuals at the Erdaojingzi site (WLR_LN in Ning et al., 2020), related to the Lower Xiajiadian culture, included B5b1a, A22, N9a1, which were all absent from the individuals at the Dongmen of Shimao City in our study. Stone carvings found in Shimao City (such as, in the center palace of Huangchengtai and Dongmen) were also observed in the Xinglongwa (∼8,200–7,400 BP) and Zhaobaogou (∼7,350–6,420 BP) culture related sites in Inner Mongolia (Lv, 1960; Wang, 1993; Sun and Shao, 2020). We didn’t find a closely maternal affinity between the populations in Dongmen and Inner Mongolia; however, more ancient genomes from Inner Mongolia are needed to confirm the genetic affinity between them.

We then focused on the genetic affinity between Shimao-related populations and the ancient populations in other regions of the YR. Our results found that the Shimao-related populations in the LN Longshan period shared more genetic relationships with the contemporaneous LN populations in the YR region, rather than those from earlier periods. Among these YR populations in the LN Longshan period, the populations related to Shimao culture showed the closest affinity with the populations related to the Taosi culture in the Middle YR’s southern Shanxi Province. Similarly, Xu (2014) conducted comparative studies of jade burial, painting and violence excavated from Shimao and Taosi cities, and showed that there were similarities in culture, and interaction in the economy, culture, and populations in these two vital cities (Xu, 2014), which were in accordance with our new findings.

Finally, we found that the LN Longshan Shimao-related populations in northern Shaanxi were closer to the present-day Han Chinese (especially the northern Han Chinese population) than to the minorities in China. A previous genetic study found that the MN Qingtai population also contributed to the Han in northern China. We found that northern Han Chinese populations shared more affinity with our Shimao-related populations than with the ancient Qingtai individuals. According to the recent census reports of the Chinese population and related historical studies, most of the humans in present-day northern Shaanxi were Han Chinese (Zhou, 2015; National Bureau of Statistics, 2021). Meanwhile, the genetic research on Han Chinese populations showed that the Han in northern China were identical and that the north-south genetic divergence was the major difference among Han populations (Li et al., 2019; He et al., 2022). Thus, our results revealed the close genetic affinities between the MN Yangshao (4,830–4,530 cal BP) and LN Longshan (4,231–3,253 cal BP) populations in northern Shaanxi Province and that they contributed to the present-day northern Han populations to a certain extent.

In summary, we conclude that the Shimao-related populations in the LN Longshan period are closely related to their predecessors in the MN Yangshao period in northern Shaanxi Province, revealing a mostly local origin for Shimao City. In addition, compared to other LN populations, the Shimao-related populations were genetically closer to the contemporaneous Taosi population in southern Shanxi Province, reflecting strong interactions in both regions of the Middle YR in the LN. As for their relationship with present-day people, the Shimao-related populations were genetically closer to northern Han population. Our study might provide a perspective for understanding the genetic affinities and population dynamics of Shimao-related populations in the Middle YR basin during the Neolithic period. Further studies with ancient genomic data will aim to test for more complex patterns of admixture and social organization in this region.

## Data Availability

The datasets presented in this study can be found in online repositories. The names of the repository/repositories and accession number(s) can be found below: Genome Warehouse in National Genomics Data Center (National Genomics Data Center Members and Partners (CNCB-NGDC), 2020; Chen et al., 2021), Beijing Institute of Genomics (China National Center for Bioinformation), Chinese Academy of Sciences, accession number PRJCA009290 (https://bigd.big.ac.cn/gwh). Other datasets for this study can be found in the Supplementary Material.

## Abbreviations

BP, before present; CWE, central-west Eurasians; DM, Dongmen; HCT, Huangchengtai; LN, late Neolithic; MN, middle Neolithic; MZZL, Muzhuzhuliang; mtDNA, Mitochondrial DNA; NC, Neicheng; NEAs, Northeastern Asians; PCA, principal component analysis; SEAs, Southeastern Asians; SGDL, Shengedaliang; TS, Taosi; XH, Xinhua; YR, Yellow River; ZS, Zhaishan; ZJZ, Zhoujiazhuang.
