# Supplementary material for: Ancient Mitogenomes Reveal the Origins and Genetic Structure of the Neolithic Shimao Population in Northern China
Source: Front Genet. 2022 May 27;13:909267. doi: 10.3389/fgene.2022.909267 (PMC9185412; doi:10.3389/fgene.2022.909267)
Supplement: Supplementary file 2 [file DataSheet1.pdf]

frontiers

Supplementary Material

1. Supplementary Data

1.1 Description of 172 newly reported ancient individuals from 13 archaeological sites

1.1.1 Sites from northern Shaanxi Province

1.1.1.1 Wuzhuangguoliang

The Wuzhuangguoliang (WZGL) site is in Xiaojie Village, Huanghaojie Township, Jingbian County, Yulin City, northern Shaanxi Province. In 2001, the Shaanxi Academy of Archaeology excavated 21 housing sites, 88 ash pits, two pottery kilns, and three tombs from the late Yangshao period to the early Longshan period. Abundant relics are unearthed at this site, including potteries, stone tools, bones, and jades (Sun et al., 2011). The maturely polished stone tools reflect developed agriculture, and the existence of chipped stone tools and microliths further confirmed the subsistence of the combination of farming, fishing, and hunting (Shi, 2012). It is worth mentioning that a large number of extremely complete animal bones are unearthed from this site (Sun et al., 2001). This site is dated to the late Yangshao and the early Longshan period (~4,950-4,450 years before present, BP) based on the archaeological evidence (Sun et al., 2011). Some relics are close to the Haishengbulang culture in the southern-central Inner Mongolia (equivalent to the late Yangshao period), while some are close to the Ashan culture (equivalent to the early Longshan period) (Sun et al., 2011). The research of physical anthropology shows that the ancient residents of the WZGL site are the closest to the East Asia types of modern Asian Mongoloid in terms of ethnic classification, and are also related to the types of Northeast Asia and South Asia (Zhou, 2012). The individuals WZGL_AH5_2sk and WZGL_BH23_2sk from the WZGL are directly radiocarbon dated to 4,832-4,622 cal BP and 4,820-4,530 cal BP, which is assigned to the Yangshao period.

1.1.1.2 Miaoliang

The Miaoliang (ML) site is located in Yangqiaopan Town, Jingbian County, Yulin City, northern Shaanxi Province. Daily necessities, production tools, and decorations made of potteries, stones, and bones are some of the artifacts unearthed. The phenomenon of burying in ash pits is a notable treatment of the dead. (Shao et al., 2019a; Shao et al., 2019b). Based on the archaeological evidence, the site may have extended from the late Yangshao period to the early Longshan period. The archaeological evidence indicated that a part of the remains of the ML site during the Yangshao period may belong to the early development stage of the Shimao culture (Shao, 2019). The individual ML_H26_2 was directly radiocarbon dated to 4,836-4,625 cal BP, which was assigned to the Yangshao period.

1.1.1.3 Shimao City

The Shimao site, also called "Shimao City", is located in Gaojiabu Town, Shenmu County, Yulin City, northern Shaanxi Province. It is a triple-structured city, taking the Huangchengtai as the core, which means the platform of the imperial city, and was surrounded by the inner city, and the outer city half surrounds the inner city. According to the radiocarbon data and archaeological evidence, Shimao City was built around 4,250 BP and was abandoned around 3,750 BP, covering an area of

over 4 million square meters. It is one of the largest known town sites in northern China, from the late Neolithic (Longshan period) to the early Bronze Age (Xia Dynasty). Shimao City is one of the biggest central settlements in the late Longshan period in northern China, with a large scale, clear structure, and complete chronological sequence (Sun et al., 2020a). And it is considered to be one of the most important ancient sites in China, which has caused great concern about the origin and developments of Chinese civilizations and early states (Sun et al., 2020a).

The ages of different sites in the Shimao City are not completely consistent, showing a trend of gradual diffusion from the Huangchengtai to the outer city. Take the sites involved in our study as an example, the Huangchengtai in the center and the Hanjiagedan, the Houyangwan, and the Mahuangliang in the inner city of the Shimao City were early, about 4,250-4,050 BP, while the Dongmen site of the outer city is later, about 4,050-3,750 BP (Sun et al., 2020a).

(1) Huangchengtai

The Huangchengtai site (HCT) is the core distribution area of the Shimao City. Enormous palaces and high-grade building sites with complex structures were found in this area as mentioned before. Some "luxury goods" like jades, stone models, murals symbolizing identity level, as well as the excavation of production relics such as copper casting and bone making, have become important evidence to infer that the HCT is the core area where high-level nobles or kings live. These relics show the HCT is not only a high-level aristocratic settlement but also a religious center. In addition, the culture of the northwest may influence the appearance of stone carvings of the HCT, according to archaeological studies (Guo, 2013; Li, 2017). Among 12 individuals from the HCT, Shimao_HCT_II3_2 and Shimao_HCT_nS3_1 are directly radiocarbon dated to 4,148-3,984 cal BP and 4,082-3,895 cal BP.

(2) Hanjiagedan

The Hanjiagedan site (HJGD) is located on an oval hill in the southeast of the HCT. In 2014, the Shimao archaeological team excavated the HJGD site and found 31 house sites, 41 tombs, 27 ash pits, four ditches, and one kiln site (Sun et al., 2016). Thousands of relics were unearthed, including potteries, stones, and bones. It is both a residential and burial site in the inner city of Shimao City. The function of the HJGD site changed. It was used as a residential site in the early stage but was abandoned and used as a cemetery later. Although the cemetery was seriously robbed and disturbed, it can still be judged from the scale that the site was a large noble cemetery in Shimao City. There are identity differences and hierarchical differentiation among the cemetery owners, and the tendency of social complexity is intensified. In addition, the phenomenon of martyrdom exists in some tombs, which may reflect the social state of class differentiation and frequent wars (Sun et al., 2020a). Two individuals (Shimao_HJGD_M21S and Shimao_HJGD_M24h) are dated to the Longshan period, 3,835-3,699 cal BP and 3,977-3,849 cal BP.

(3) Houyangwan

The Houyangwan site (HYW) is in the northeast of the HCT site. It is also a residential area in the inner city of Shimao City, similar to the HJGD. In 2012, trial excavations for the HYW were conducted. House sites and tombs are the main relics of this site. The types of house sites are cave-dwelling. And the tombs include vertical caves, earth pit tombs, and urn coffin tombs. The unearthed relics are mainly potteries, besides a few stone tools, bone vessels, and many animal bones. It is

worth noting that some tombs have the phenomenon of martyrdom (Sun et al., 2015; Sun et al., 2020a). Two individuals, Shimao_HYW_T1M2b and Shimao_HYW_T2M2, are directly radiocarbon dated to 3,975-3,840 cal BP and 3,811-3,570 cal BP, which are also assigned to the Longshan period.

(4) Mahuangliang

The Manghuangliang site (MHL) is located in the inner city of the Shimao City. There is only one individual in our analysis, which is directly radiocarbon dated to 3,894-3,722 cal BP.

(5) Dongmen

The Dongmen site (DM) is located in the northeast of the outer city. It is a prehistoric gate site with a distinct structure and exquisite design in China. Important relics such as jade wares, potteries, murals, and stone carving anthropopathic features have been unearthed in the surrounding layers. The jades were found in the stone walls, which may have the religious function of warding off evil spirits. A total of six sacrifice pits with human skulls were found on this site. These skulls, buried intensively, are located under the early ground or stone wall. The research in physical anthropology shows that there are more women than men and no minors in these pits. From the perspective of species characteristics, they are highly consistent with the indigenous people in the pre-Qin period along the great wall of Inner Mongolia (Chen et al., 2016). It is likely to be related to the foundation laying or sacrificial activities during the construction of the city wall (Shao, 2016; Sun et al., 2020a). The radiocarbon dating of individual Shimao_DM_K4_8, Shimao_DM_M2, and Shimao_DM_K6_2 are 4,144-3,976 cal BP, 3,390-3,253 cal BP and 4,084-3,902 cal BP.

1.1.1.4 Shengedaliang

The Shengedaliang site (SGDL) is located in Yejihe Village, Dabaodang Town, Shenmu County, Yulin City, northern Shaanxi Province. Many remains, such as tombs, ash pits, house foundations, and rammed-earth foundation, were unearthed in 2013-2014. Substantial artifacts dating to the Longshan and the Xia periods were unearthed, including potteries, stone tools, bone artifacts, and other relics. The combination of artifacts unearthed from this site is basically as same as those found in the Xinhua site (XH), the Shimao City, and the Muzhuzhuliang site (MZZL) (Guo et al., 2016). Individuals SGDL_M7_2014, SGDL_M17, SGDL_M14 and SGDL_M25 are directly radiocarbon dated to 3,811-3,570 cal BP, 3,868-3,650 cal BP, 3,959-3,728 cal BP, and 3,969-3,831 cal BP, which are assigned to the Longshan period.

1.1.1.5 Muzhuzhuliang

The Muzhuzhuliang site (MZZL) is located in Yejihe Village, Dabaodang Town, Shenmu County, Yulin City, northern Shaanxi Province. Plenty of house sites, ash pits were discovered with a few tombs, pottery kilns, ditches, and other relics. The unearthed relics were very similar to those on the SGDL site. This site is considered to be the most complete settlement with circular moats in the late Longshan period in northern Shaanxi (Guo, 2015; Wang et al., 2015). The research of physical anthropology research shows that the ancient population of the MZZL is close to the East Asian type of Mongoloid, and skull characteristics show they are similar to the characteristics of the ancient population of the Miaozigou, which locates in the south-central Inner Mongolia (Chen et al., 2015). The radiocarbon dating of MZZL_H32, MZZL_M3 and MZZL_M7 are also the Longshan period, 4,082-3,895 cal BP, 3,966-3,727 cal BP and 3,964-3,722 cal BP.

1.1.1.6 Xinhua

The Xinhua site (XH) locates in Xinhua Village, Dabaodang Town, Shenmu County, northern Shaanxi Province. A total of 155 ash pits, 72 tombs, 33 house sites, five kiln sites, and one jade pit and abundant artifacts (such as potteries, stone tools, bones, and jades) were unearthed (Sun, 2005; Xing et al., 2005). It is worth mentioning that 32 pieces of jade were unearthed in K1, which is considered a sacrificial pit (Sun, 2002). The radiocarbon dating data of individuals XH_M1b, XH_M58, and XH_M59 are 3,835-3,652 cal BP, 3,868-3,696 cal BP, and 4,231-3,998 cal BP, respectively.

1.1.1.7 Zhaishan

The Zhaishan site (ZS) is in Wangshamao Village, Tianjiazhai Town, Fugu County, northern Shaanxi Province, 60 kilometers away from Shimao City in the southwest. The site contains a stone city settlement dated to the Longshan period, covering an area of about 600,000 square meters. The rammed earth platform found in the north of the city and wrapped with the stone wall, which is similar to the structure of Shimao City. Therefore, the platform may be the core area of ZS stone city. Besides, ZS was considered to be the Shimao culture due to the similarities of the artifacts. 21 tombs with obvious hierarchy were found. The tombs excavated at ZS can be divided into different hierarchies according to the scale of tombs, the number of funerary objects, the existence of burial utensils, and martyrs, which shows that class differentiation has existed among the populations in this settlement (Sun et al., 2020b; Shao, 2021). And the site is considered to be in the Longshan period, around 4,050-3,750 BP (Shao et al., 2021).

1.1.2 Site from northern Shanxi Province

1.1.2.1 Taosi

The Taosi site (TS) locates in the south of Taosi Villiage, Xiangfen County, Linfen City in Shanxi Province, covering an area of 2.8 million square meters. It is one of the largest Longshan cultural sites in the Central Plain, dated from 4300 to 3900 BP according to the radiocarbon data and archaeological evidence, which contains three periods: early (~4,300-4,100 BP), middle (~4,100-4,000 BP) and late period (~4,000-3,900 BP). Based on the characteristics of the remains of the TS, archaeologists regarded the Taosi culture as another new type of the Longshan Culture in the Middle YR (Gao, 1980; He, 2004; Yan and He, 2005). The similarities between the Shimao City and the Taosi site in jade, color painting, and acts of violence show that there is close interaction and connection between the two regions (Xu, 2014). The research of physical anthropology shows that the morphological characteristics of human bones in the Longshan period are between East Asian and South Asian Mongoloid (Zhang, 2009). Among three individuals from TS, one individual, TS_G33, is directly radiocarbon dated to 3,869-3,697 cal BP, the Longshan period.

1.1.2.2 Zhoujiazhuang

The Zhoujiazhuang site (ZJZ) is located in Zhoujiazhuang Village, Hengshui Town, Jiangxian County, Shanxi Province. ZJZ has a large period, including the relics of Yangshao, Miaodigou period II, Longshan, Erlitou, Erligang, Zhou, and Han dynasties. Among them, the remains of the Longshan period are the most widely distributed, covering an area of 4.5 million square meters. According to the archaeological study, the overall characteristics of the artifacts are close to the Taosi site (Dai et al., 2018; Tian and Dai, 2018). The 37 individuals from the ZJZ are considered to be in the Longshan period, 4,150-3,700 BP (Sun, 2018).

1.2 Groupings for the newly reported ancient individuals

We collected five individuals from the ML and 16 individuals from the WZGL. These two sites are showed similarities in their geographical locations, dates, and excavated relics, and we grouped these 21 individuals as "preShimao_MW".

A total of 71 individuals were obtained from the Shimao City. We grouped them based on their dates and locations in Shimao City: The "Shimao_HCT" group contained 10 individuals collected from the HCT, which was in the center of the Shimao City and had the highest political status. We grouped the HJGD, the HYW, and the MHL in the inner city of the Shimao City as "Shimao_NC "(n=48) for their similar date, location, and relics. For the 13 individuals from the Dongmen site of Shimao's outer city, we named as "Shimao_DM" group.

For the other Shimao-related individuals, we grouped them as follows. The 12 individuals from the SGDL and six individuals from the MZZL were grouped as "MZZSGDL" for their close geographical distances and similar date. The 12 and 10 individuals from the XH and the ZS were grouped as "XH" and "ZS", respectively. As for the individuals from Shanxi, we grouped the three individuals from the TS and 37 individuals from the ZJZ as "TSZJZ" for their similar dates, locations, and excavated relics.

There are three pairs of kinship (the same mitochondrial sequences) among the individuals from the HJGD (Shimao_HJGD_M6S and Shimao_HJGD_M26S, Shimao_HJGD_M34S and Shimao_HJGD_M34h, Shimao_HJGD_M36O and Shimao_HJGD_M36h), and one pair of kinship among the individuals from the XH (XH_M4a and XH_M24). We excluded four individuals, Shimao_HJGD_M6S, Shimao_HJGD_M34S, Shimao_HJGD_M36O, and XH_M24, for their relatively lower coverage in these four pairs of kinship. Besides, because of the high contamination (> 4%), we also excluded six individuals, which are Shimao_HYW_T2M2 from the HYW (8.0%), Shimao_DM_K4_10_2 from the DM (9.5%), MZZL_M7 and MZZL_M8 from the MZZL (6.8% and 5.1%, respectively), and XH_M1a and XH_M48 from the XH (10.2% and 14.0%, respectively). Therefore, there were 44 individuals in the Shimao_NC group, 12 individuals in the Shimao_DM group, four individuals in the MZZL, and nine individuals in the XH group. In summary, we used 162 individuals in these groups for further analysis.

1.3 Published individual classification and nomenclature

Our dataset includes 801 ancient individuals and 7,641 present-day individuals (**Table S1**). These individuals are grouped into four clusters according to the PCA based on haplogroup frequency and F_{ST} heatmap based on genetic distance: North-eastern Asian (NEA: North Asians and Northern East Asians), South-eastern Asian (SEA: South Asian and Southern East Asians), Central and West Eurasian (CWE: Central and West Asian including populations from Xinjiang, China, and European populations).

1.3.1 Classification of ancient samples from NEA.

The PCA results show populations from northern China (north of the Qinling-Huaihe Line) and populations from North Asia (Baikal_EN, Baikal_EBA, N.Mongolia_LBA, Xiongnu_HP) gathered together and located far away from the populations in southern China (**Figure 2B**). Therefore, we put the samples from northern China, Mongolia, and the Baikal Lake basin into one group called North-eastern Asia (NEA). Although the individuals from Xinjiang are located in northern China, the genetic analyses show that the individuals in different regions and periods showed different genetic

structures, we grouped the individuals following the genetic results (Wang et al., 2021). Therefore, we divided 224 samples from Xinjiang and 220 from Central and Western Asian samples into one group, named Central and Western Eurasia (CWE).

We grouped 473 samples from northern China based on the archaeological culture, date, and geographical location. 74 individuals are from the Gansu-Qinghai region and Tibet, including 15 individuals from the areas above 4,000 meters (Ding et al., 2020). For the samples with the age range of 3,150-511 BP, we followed the study of Ding et al. and grouped them with seven ancient individuals from high valley of the Himalayan arc in Nepal into HTP_IA (Jeong et al., 2016; Ding et al., 2020). To explore the samples from the YR basin further, we grouped LTP_IA populations finely according to archaeological culture and geographical location. For the individuals in the Gan-Qing region, we grouped the 29 individuals of the Majiayao culture with an average age of 3,957 BP (range 5,040-411 BP) together, named GQMajiaY_EBA. 11 individuals belonging to the Qijia culture with an average age of 3,351 BP (range 4,065-1,791 BP) were divided into one group named GQQijia_BA, and eight individuals of the Kayue culture with the date ranging from 2,500BP to 3,200BP, were named GQKayue_LBA as one group. Besides, for the 11 individuals in some areas of Tibet with an altitude of lower than 4,000 meters, we grouped them as LTP_IA.

In the Middle YR, in addition to the 40 TSZJZ samples from southern Shanxi we analyzed, there are 52 samples from the Qingtai site in Henan (Miao et al., 2021). Since these samples are from the same site and dated to the Yangshao period within 5,500-5,000 BP, we divided them into a group and named them QT_MN.

In the Lower YR, 87 samples were obtained of 9,500-1,800 BP in Shandong (Liu J et al., 2021). According to the current research, the genetic composition of Shandong populations has changed around 4,600 BP (Liu J et al., 2021). Therefore, we divided 50 individuals after 4,600 BP into one group called SD_LN refer to Liu J et al. Besides, for the individuals before 4,600BP, we observed that five earlier individuals from the Bianbian, the Xiaogao, and the Xiaojingshan belong to the early Neolithic period (~9,500-7,000 BP), while the other 37 samples belong to the middle Neolithic period (~6,000-4,600 BP). In addition, the five earlier individuals have the haplotypes N9a2'4'5'11, B4c1c, and D4b2b2 that are not carried by other Shandong individuals. Therefore, we divided the five individuals into one group named SD_EN and the other 37 individuals named SD_MN.

Besides, we also obtained haplogroup information of 36 individuals from Halahaigou site in Inner Mongolia. The individuals are dated to ~4,500 BP and belong to the middle Neolithic period (Zhao, 2009). Therefore, we named them as Halahaogou_MN.

1.3.2 Classification of ancient samples from other regions.

We obtained 59 individuals in southern China (south of Qinling-Huaihe) and grouped them into four groups (FJ_LN, 11 individuals; YN_LN five individuals; YNHC_LN, 11 individuals; GX_HE, 32 individuals) refer to Liu Y et al. (Liu Y et al., 2021). In addition, 22 individuals of HTP_IA are also located in the south of China, and the frequency of haplogroup M is the highest same as the above four groups in southern China. As a result, we put these five groups within the South Asian populations.

For the 220 individuals in the CWE, we followed the grouping of Wang et al. and divided them into 16 populations (Wang et al., 2021).

1.3.3 Classification of present-day individuals

We collected 7,641 present-day individuals from East Asia, South Asia, North Asia, and the Central and Western Eurasia (Table S1), and mainly grouped them by their geographical locations. It was also grouped according to the grouping of Wang et al. and divided them into 26 populations except for the individuals from China (Wang et al., 2021).

As for the 2,102 present-day samples from China, we grouped them more finely. They were grouped mainly based on their geographical location and nationality. Since the Han is the largest nationality in China and distributes widely, we grouped the Han individuals into the Northern Han (NChina_Han) and the Southern Han (SChina_Han) (Liu Y et al., 2021). The NChina_Han includes 388 samples from Beijing, Ningxia, Liaoning, Shandong, Shaanxi, Gansu, and Xinjiang Province, while the SChina_Han contains 168 samples from Hubei, Hunan, Guangdong, Guangxi, and Yunnan Province. For ethnic minorities in northern China, we grouped them according to their nationalities. Besides, individuals in southern China, except for the Han, were grouped based on the geographical location and nationality comprehensively.

Reference

Allentoft, M.E., Sikora, M., Sjögren, K.G., Rasmussen, S., Rasmussen, M., Stenderup, J., et al. (2015). Population genomics of Bronze Age Eurasia. Nature. 522(7555), 167-172. doi: 10.1038/nature14507

Bhandari, S., Zhang, X., Cui, C., Bianba, Liao, S., Peng, Y., Zhang, H., et al. (2015). Genetic evidence of a recent Tibetan ancestry to Sherpas in the Himalayan region. Scientific reports. 5, 16249. doi: 10.1038/srep16249

Brandão, A., Eng, K.K., Rito, T., Cavadas, B., Bulbeck, D., Gandini, F., et al. (2016). Quantifying the legacy of the Chinese Neolithic on the maternal genetic heritage of Taiwan and Island Southeast Asia. Human genetics. 135(4), 363-376. doi: 10.1007/s00439-016-1640-3

Cann, H.M., de Toma, C., Cazes, L., Legrand, M.F., Morel, V., Piouffre, L., et al. (2002). A human genome diversity cell line panel. Science. 296(5566), 261-262. doi: 10.1126/science.296.5566.261b

Chandrasekar, A., Kumar, S., Sreenath, J., Sarkar, B. N., Urade, B.P., Mallick, S., et al. (2009). Updating phylogeny of mitochondrial DNA macrohaplogroup m in India: dispersal of modern human in South Asian corridor. PloS one. 4(10), e7447. doi: 10.1371/journal.pone.0007447

Chen, L., Guo, X.N., Hong, X.Y., and Wang, W.L. (2015). The analysis of human skeletons from Muzhuzhuliang site, Shenmu County, Shaanxi Province. Archaeology and Cultural Relics. (05), 118-123.

Chen, L., Xiong, J.X., Shao, J., and Sun, Z.Y. (2016). The research of the forensic analysis of skulls from the sacrifical pits located in Shimao site, Shaanxi Province. Archaeology and Cultural Relics. (04), 134-142.

Dai, X.M., Tian, W., Wang, W.W., and Lv, Z.L. (2018). A Report of Excavation from 2007 to 2013 of Zhou Remains at Zhoujiazhuang. Journal of National Museum of Chinese History. (03), 6-20.

Damgaard, P.B., Martiniano, R., Kamm, J., Moreno-Mayar, J.V., Kroonen, G., Peyrot, M., Barjamovic, G., et al. (2018a). The first horse herders and the impact of early Bronze Age steppe expansions into Asia. Science. 360(6396), eaar7711. doi: 10.1126/science.aar7711

Damgaard, P.B., Marchi, N., Rasmussen, S., Peyrot, M., Renaud, G., Korneliussen, T., et al. (2018b). 137 ancient human genomes from across the Eurasian steppes. Nature. 557(7705), 369-374. doi: 10.1038/s41586-018-0094-2

Derenko, M., Malyarchuk, B., Bahmanimehr, A., Denisova, G., Perkova, M., Farjadian, S., et al. (2013). Complete mitochondrial DNA diversity in Iranians. PloS one. 8(11), e80673. doi: 10.1371/journal.pone.0080673

Derenko, M., Malyarchuk, B., Denisova, G., Perkova, M., Litvinov, A., Grzybowski, T., et al. (2014). Western Eurasian ancestry in modern Siberians based on mitogenomic data. BMC evolutionary biology. 14, 217. doi: 10.1186/s12862-014-0217-9

Derenko, M., Malyarchuk, B., Denisova, G., Perkova, M., Rogalla, U., Grzybowski, T., et al. (2012). Complete mitochondrial DNA analysis of eastern Eurasian haplogroups rarely found in populations of northern Asia and eastern Europe. PloS one. 7(2), e32179. doi: 10.1371/journal.pone.0032179

Derenko, M., Malyarchuk, B., Grzybowski, T., Denisova, G., Dambueva, I., Perkova, M., et al. (2007). Phylogeographic analysis of mitochondrial DNA in northern Asian populations. American journal of human genetics. 81(5), 1025-1041. doi: 10.1086/522933

Derenko, M., Malyarchuk, B., Grzybowski, T., Denisova, G., Rogalla, U., Perkova, M., et al. (2010). Origin and post-glacial dispersal of mitochondrial DNA haplogroups C and D in northern Asia. PloS one. 5(12), e15214. doi: 10.1371/journal.pone.0015214

Ding, M.Y., Wang T.Y., Ko, A.M., Chen, H.H., Wang, H., and Dong, G.H. (2020). Ancient mitogenomes show plateau populations from last 5200 years partially contributed to present-day Tibetans. Proceedings. Biological sciences. 287(1923), 20192968. doi: 10.1098/rspb.2019.2968.

Duggan, A.T., Whitten, M., Wiebe, V., Crawford, M., Butthof, A., Spitsyn, V., et al. (2013). Investigating the Prehistory of NChina_Tungusic Peoples of Siberia and the Amur-Ussuri Region with Complete mtDNA Genome Sequences and Y-chromosomal Markers. Plos One. 8(12), e83570. doi: 10.1371/journal.pone.0083570

Duong, N.T., Macholdt, E., Ton, N.D., Arias, L., Schröder, R., Van Phong, N., et al. (2018). Complete human mtDNA genome sequences from Vietnam and the phylogeography of Mainland Southeast Asia. Scientific reports. 8(1), 11651. doi: 10.1038/s41598-018-29989-0

Fendt, L., Zimmermann, B., Daniaux, M., and Parson, W. (2009). Sequencing strategy for the whole mitochondrial genome resulting in high quality sequences. BMC genomics. 10, 139. doi: 10.1186/1471-2164-10-139

Fernandes, V., Alshamali, F., Alves, M., Costa, M.D., Pereira, J.B., Silva, N.M., et al. (2012). The Arabian cradle: mitochondrial relicts of the first steps along the southern route out of Africa. American journal of human genetics. 90(2), 347-355. doi: 10.1016/j.ajhg.2011.12.010

Finnilä, S., Lehtonen, M.S., and Majamaa, K. (2001). Phylogenetic network for European mtDNA. American journal of human genetics. 68(6), 1475-1484. doi: 10.1086/320591

Fornarino, S., Pala, M., Battaglia, V., Maranta, R., Achilli, A., Modiano, G., et al. (2009). Mitochondrial and Y-chromosome diversity of the Tharus (Nepal): a reservoir of genetic variation. BMC evolutionary biology. 9, 154. doi: 10.1186/1471-2148-9-154

Fraumene, C., Belle, E.M., Castrì, L., Sanna, S., Mancosu, G., Cosso, M., Marras, F., Barbujani, G., Pirastu, M., & Angius, A. (2006). High resolution analysis and phylogenetic network construction using complete mtDNA sequences in sardinian genetic isolates. Molecular biology and evolution. 23(11), 2101 – 2111. doi: 10.1093/molbev/msl084

Gao, T.L., and Zhao, D.H. (1980). Archaeological survey of excavation at Taosi site, Xiangfen County, Shanxi Province. Archaeology. (01), 18-31+100-102.

Gunnarsdóttir, E.D., Li, M., Bauchet, M., Finstermeier, K., and Stoneking, M. (2011). High-throughput sequencing of complete human mtDNA genomes from the Philippines. Genome research. 21(1), 1-11. doi: 10.1101/gr.107615.110

Guo, W. (2013). The communication between northern China and Eurasian steppe during the Longshan period based on the stone carving from the Shimao site. http://kaogu.cn/html/cn/xueshuyanjiu/yanjiuxinlun/juluoyuchengshikaog/2013/1025/33681.html [Accessed March 30, 2022].

Guo, X.N., (2015). Stages of the Muzhuzhuliang site in Shenmu County. Archaeology and Cultural Relics. (05), 32-36.

Guo, X.N., Wang, W.L., Kang, N.W., Qu, F.M., and Chen, L. (2016). The excavation report of Shengedaliang site, Shenmu county, Shaanxi province. Archaeology and Cultural Relics. (04), 34-44+145.

He, G.L., Wang, M.G., Li, Y.X., Zou, X., Yeh, H.Y., Tang, R.K., Yang, X.M., Wang, Z., Guo, J.X., Luo, T., Zhao, J., Sun, J., Hu, R., Wei, L.H., Chen, G., Hou, Y.P., and Wang, C.C. (2022). Fine-scale north-to-south genetic admixture profile in Shaanxi Han Chinese revealed by genome-wide demographic history reconstruction. J. Syst. Evol. doi: 10.1111/jse.12715

He, N. (2004). Review on the research history of Taosi culture. Ancient Civilization. 3(00), 54-86.

Ingman, M., and Gyllensten, U. (2007). Rate variation between mitochondrial domains and adaptive evolution in humans. Human molecular genetics, 16(19), 2281-2287. doi: 10.1093/hmg/ddm180

Jeong, C., Ozga, A.T., Witonsky, D.B., Malmström, H., Edlund, H., Hofman, C.A., et al. (2016). Long-term genetic stability and a high-altitude East Asian origin for the peoples of the high valleys of the Himalayan arc. Proc Natl Acad Sci USA. 113(27), 7485-7490. doi: 10.1073/pnas.1520844113

Jeong, C., Wilkin, S., Amgalantugs, T., Bouwman, A. S., Taylor, W., Hagan, R.W., et al. (2018). Bronze Age population dynamics and the rise of dairy pastoralism on the eastern Eurasian steppe. Proc Natl Acad Sci USA. 115(48), E11248-E11255. doi: 10.1073/pnas.1813608115

Kang, L., Zheng, H.X., Zhang, M., Yan, S., Li, L., Liu, L., Liu, K., et al. (2016). MtDNA analysis reveals enriched pathogenic mutations in Tibetan highlanders. Scientific reports. 6, 31083. doi: 10.1038/srep31083

Ko, A.M., Chen, C.Y., Fu, Q., Delfin, F., Li, M., Chiu, H.L., Stoneking, M., and Ko, Y.C. (2014). Early Austronesians: into and out of Taiwan. American journal of human genetics. 94(3), 426-436.

Kong, Q.P., Sun, C., Wang, H.W., Zhao, M., Wang, W.Z., Zhong, L., et al. (2011). Large-scale mtDNA screening reveals a surprising matrilineal complexity in east Asia and its implications to the peopling of the region. Molecular biology and evolution. 28(1), 513-522. doi: 10.1093/molbev/msq219

Kong, Q.P., Yao, Y.G., Sun, C., Bandelt, H. J., Zhu, C.L., and Zhang, Y.P. (2003). Phylogeny of east Asian mitochondrial DNA lineages inferred from complete sequences. American journal of human genetics. 73(3), 671-676. doi: 10.1086/377718

Kushniarevich, A., Sivitskaya, L., Danilenko, N., Novogrodskii, T., Tsybovsky, I., Kiseleva, A., et al. (2013). Uniparental genetic heritage of belarusians: encounter of rare middle eastern matrilineages with a central European mitochondrial DNA pool. PloS one. 8(6), e66499. doi: 10.1371/journal.pone.0066499

Kutanan, W., Kampuansai, J., Srikummool, M., Kangwanpong, D., Ghirotto, S., Brunelli, A., et al. (2017). Complete mitochondrial genomes of Thai and Lao populations indicate an ancient origin of Austroasiatic groups and demic diffusion in the spread of Tai-Kadai languages. Human genetics. 136(1), 85-98. doi: 10.1007/s00439-016-1742-y

Li, M. (2017). Coming back to Xiaxu--Social memory and the emergence of classial tradition. Acta Archaeological Sinica. (03), 287-316

Li, Y.C., Wang, H.W., Tian, J.Y., Liu, L.N., Yang, L.Q., Zhu, C.L., et al. (2015). Ancient inland human dispersals from Myanmar into interior East Asia since the Late Pleistocene. Scientific reports. 5, 9473. doi: 10.1038/srep09473

Liu, J.C., Zeng, W., Sun, B., Mao, X.W., Zhao, Y.S., Wang, F., et al. (2021). Maternal genetic structure in ancient Shandong between 9500 and 1800 years ago. Science Bulletin. (11), 1129-1135. doi: 10.1016/J.SCIB.2021.01.029.

Liu, Y.L., Wang, T.Y., Wu, X.C., Fan, X.C., Wang, W., Xie, G.M., et al. (2021). Maternal genetic history of southern East Asians over the past 12,000 years. J Genet Genomics. 48(10), 899-907. doi:10.1016/j.jgg.2021.06.002

Maca-Meyer, N., González, A.M., Larruga, J.M., Flores, C., and Cabrera, V.M. (2001). Major genomic mitochondrial lineages delineate early human expansions. BMC genetics. 2, 13. doi: 10.1186/1471-2156-2-13

Malyarchuk, B., Derenko, M., Denisova, G., and Kravtsova, O. (2010). Mitogenomic diversity in Tatars from the Volga-Ural region of Russia. Molecular biology and evolution. 27(10), 2220-2226. doi:10.1093/molbev/msq065

Malyarchuk, B., Derenko, M., Denisova, G., Litvinov, A., Rogalla, U., Skonieczna, K., et al. (2018). Whole mitochondrial genome diversity in two Hungarian populations. Molecular genetics and genomics. 293(5), 1255-1263. doi: 10.1007/s00438-018-1458-x

Malyarchuk, B., Grzybowski, T., Derenko, M., Perkova, M., Vanecek, T., Lazur, J., et al. (2008). Mitochondrial DNA phylogeny in Eastern and Western Slavs. Molecular biology and evolution, 25(8), 1651-1658. doi: 10.1093/molbev/msn114

Miao, B., Liu Y.C., Gu, W.F., W, Q.L., Wu, Q., Wang, W.J., et al. (2021). Maternal genetic structure of a neolithic population of the Yangshao culture. Journal of Genetics and Genomics. (08), 746-750. doi: 10.1016/j.jgg.2021.04.005

Narasimhan, V.M., Patterson, N., Moorjani, P., Rohland, N., Bernardos, R., Mallick, S., et al. (2019). The formation of human populations in South and Central Asia. Science. 365(6457), eaat7487. doi: 10.1126/science.aat7487

Pala, M., Olivieri, A., Achilli, A., Accetturo, M., Metspalu, E., Reidla, M., et al. (2012). Mitochondrial DNA signals of late glacial recolonization of Europe from near eastern refugia. American journal of human genetics. 90(5), 915-924. doi: 10.1016/j.ajhg.2012.04.003

Palanichamy, M.G., Zhang, C.L., Mitra, B., Malyarchuk, B., Derenko, M., Chaudhuri, T.K., et al. (2010). Mitochondrial haplogroup N1a phylogeography, with implication to the origin of European farmers. BMC evolutionary biology. 10, 304. doi: 10.1186/1471-2148-10-304

Pankratov, V., Litvinov, S., Kassian, A., Shulhin, D., Tchebotarev, L., Yunusbayev, B., et al. (2016). East Eurasian ancestry in the middle of Europe: genetic footprints of Steppe nomads in the genomes of Belarusian Lipka Tatars. Scientific reports. 6, 30197. doi: 10.1038/srep30197

Peng, M.S., Palanichamy, M.G., Yao, Y.G., Mitra, B., Cheng, Y.T., Zhao, M., et al. (2011). Inland post-glacial dispersal in East Asia revealed by mitochondrial haplogroup M9a'b. BMC biology. 9, 2. doi: 10.1186/1741-7007-9-2

Peng, M.S., Xu, W., Song, J. J., Chen, X., Sulaiman, X., Cai, L., et al. (2018). Mitochondrial genomes uncover the maternal history of the Pamir populations. European journal of human genetics. 26(1), 124-136. doi: 10.1038/s41431-017-0028-8

Pereira, L., Gonçalves, J., Franco-Duarte, R., Silva, J., Rocha, T., Arnold, C., et al. (2007). No evidence for an mtDNA role in sperm motility: data from complete sequencing of asthenozoospermic males. Molecular biology and evolution. 24(3), 868-874. doi: 10.1093/molbev/msm004

Piotrowska, N.A., Kosior, J.E., Schab, A., Wrobel, D.D., Bartnik, E., Zarnowski, T., et al. (2019). Investigation of whole mitochondrial genome variation in normal tension glaucoma. Experimental eye research. 178, 186-197. doi: 10.1016/j.exer.2018.10.004

Qin, Z.D., Yang, Y.J., Kang, L.L., Yan, S., Cho, K., Cai, X.Y., et al. (2010). A mitochondrial revelation of early human migrations to the Tibetan Plateau before and after the last glacial maximum. American journal of physical anthropology. 143(4), 555-569. doi: 10.1002/ajpa.21350

Raule, N., Sevini, F., Li, S., Barbieri, A., Tallaro, F., Lomartire, L., et al. (2014). The co-occurrence of mtDNA mutations on different oxidative phosphorylation subunits, not detected by haplogroup analysis, affects human longevity and is population specific. Aging cell. 13(3), 401-407. doi: 10.1111/acel.12186

Sahakyan, H., Hooshiar Kashani, B., Tamang, R., Kushniarevich, A., Francis, A., Costa, M. D., et al. (2017). Origin and spread of human mitochondrial DNA haplogroup U7. Scientific reports. 7, 46044. doi: 10.1038/srep46044

Schönberg, A., Theunert, C., Li, M., Stoneking, M., and Nasidze, I. (2011). High-throughput sequencing of complete human mtDNA genomes from the Caucasus and West Asia: high diversity and demographic inferences. European journal of human genetics. 19(9), 988-994. doi: 10.1038/ejhg.2011.62

Shao, J. (2016). The article synthesizes of Chronology and Constructional process of Shimao stone walled-city. Archaeology and Cultural Relics. (04), 102-108.

Shao, J. (2019). The archaeology remains of Early Longshan Era in Northern Shaanxi. Archaeology and Cultural Relics. (04), 61-65.

Shao, J. (2021). Neolithic site of Zhaishan in Fugu, Shaanxi province. Appreciation. (07), 63-65+62.

Shao, J., Di, N., Yang, G. Q., He, C.L., Wang, Y.Y., Zhao, X.H., et al., (2019a). Archaeological survey of excavation in Longshan period at Miaoliang site, Jingbian County, Shaanxi Province. Archaeology and Cultural Relics. (04), 03-11.

Shao, J., Di, N., Yang, G.Q., He, C.L., Wang, Y.Y., Zhao, X.H., et al., (2019b). Archaeological survey of excavation in Yangshao period at Miaoliang site, Jingbian County, Shaanxi Province. Wenbo. (01), 3-12+2.

Shao, J., Pei, X.S., Di, N., Yuan, Y., Zhao, K., and He, C.L. (2021). Archeology survey of excavation at Miaoyan locality in Zhaishan site in Fugu County, Shaanxi. Wenbo. (05), 15-28+2+113.

Shi, J. (2012) A study of Neolithic remains in Wuzhuangguoliang site , Jingbian County, Shaanxi Province. [dissertation/master's thesis]. [Xi'an (Shaanxi)]: Northwest University.

Shlush, L.I., Behar, D.M., Yudkovsky, G., Templeton, A., Hadid, Y., Basis, F., et al. (2008). The Druze: a population genetic refugium of the Near East. PloS one. 3(5), e2105. doi: 10.1371/journal.pone.0002105

Summerer, M., Horst, J., Erhart, G., Weißensteiner, H., Schönherr, S., Pacher, D., et al. (2014). Large-scale mitochondrial DNA analysis in Southeast Asia reveals evolutionary effects of cultural isolation in the multi-ethnic population of Myanmar. BMC evolutionary biology. 14, 17. doi: 10.1186/1471-2148-14-17

Sun, Q. W. (2018). Living within boundries of Xia. Beijing: SDX Joint publishing company.

Sun, Z.Y. (2002). The relevant questions about unearthed jades from Xinhua site, Shenmu County. Cultural Relics of Central China. (05), 37-42.

Sun, Z.Y. (2005). The review of Xinhua site. Archaeology and Cultural Relics. (03), 40-48+59.

Sun, Z.Y., Kang, L.Y., Xu, Y.C., and Qiao, J.J. (2001). There were plenty of archaeological harvest in the late Yangshao site in Jingbian, Shaanxi. Weekly of China's Cultural Relics. 002.

Sun, Z.Y., Shao. J., and Di, N. (2020a). Archaeological discovery and research synthesis of Shimao site. Cultural Relics of Central China. (01), 39-62.

Sun, Z.Y., Shao, J., and Di, N. (2020b). Nomenclature, range and age of Shimao site. Archaeology (08), 101-104+130+105-108.

Sun, Z.Y., Shao, J., Shao, A.D., Kang, N.W., Qu, F.M., and Bai, H.L. (2015). Archeology survey of excavation at Houyangwan and Hujiawa locality in Shimao site in Shenmu County, Shaanxi. Archaeology. (05), 60-71+2.

Sun, Z.Y., Xu, Y.C., Li, W.H., and Shi, J. (2011). Archeology survey of excavation at Wuzhuangguoliang site, Jingbian County, Shaanxi Province. Cultural Relics of Central China. (06), 53-63.

The 1000 Genomes Project Consortium., Auton, A., Brooks, L.D., Durbin, R.M., Garrison, E.P., Kang, H.M., et al. (2015). A global reference for human genetic variation. Nature 526, 68-74. doi: 10.1038/nature15393

Tian, W., and Dai, X.M. (2018). Archeology survey of excavation at Zhoujiazhuang site, Jiang County, Shanxi Province, in 2013. Archeology. (01), 18-31+100-102.

Wang, H.W., Li, Y.C., Sun, F., Zhao, M., Mitra, B., Chaudhuri, T.K., et al. (2012). Revisiting the role of the Himalayas in peopling Nepal: insights from mitochondrial genomes. Journal of human genetics. 57(4), 228-234. doi: 10.1038/jhg.2012.8

Wang, T.Y., Wang, W., Xie, G.M., Li, Z., Fan, X.C., Yang, Q.P., et al. (2021). Human population history at the crossroads of East and Southeast Asia since 11,000 years ago. Cell. 184(14), 3829–3841.e21. doi: 10.1016/j.cell.2021.05.018

Wang, W.J., Ding, M.Y., Gardner, J.D., Wang, Y.Q., Miao, B., Guo, W., et al. (2021). Ancient Xinjiang mitogenomes reveal intense admixture with high genetic diversity. Sci Adv. 7(14), eabd6690. doi: 10.1126/sciadv.abd6690

Wang, W.L., Guo, X.N., Kang, N.W., Liu, X.M., Hu, K., and Chen, L. (2015). Archaeology survey on the Muzhuzhuliang site in Shenmu County, Shaanxi. Archaeology and Cultural Relics. (05), 3-11+43+2+129.

Xing, F.L., Li, M., and Sun, Z.Y. (2002). Brief excavation report of Xinhua site, Shenmu, Shaanxi in 1999. Archaeology and Cultural Relics. (01), 3-12+97.

Xu, F. (2014). Preliminary comparison of Shimao and Taosi archaeology discovery. Wenbo. (01), 18-22+69.

Yan, Z.B., and He, N. (2005). Archaeology survey on the Taosi site in Xiangfen County, Shanxi. Acta Archaeologica Sinica. (03), 307-346+381-387+390.

Yao, H.B., Wang, M.G., Zou, X., Li, Y.X., Yang, X.M., Li, A.L., et al. (2021). New insights into the fine-scale history of western-eastern admixture of the northwestern Chinese population in the Hexi Corridor via genome-wide genetic legacy. Molecular genetics and genomics. 296(3), 631 – 651. doi: 10.1007/s00438-021-01767-0

Zhang, X.M., Li, C.M., Zhou, Y.N., Huang, J.H., Yu, T.S., Liu, X., et al. (2020). A Matrilineal Genetic Perspective of Hanging Coffin Custom in Southern China and Northern Thailand. iScience, 23(4), 101032. doi: 10.1016/j.isci.2020.101032

Zhang, Y.J., He, N., and Zhang, F. (2009). The Racial Type of the Middle-Later Phases of the Taosi Culture of Shanxi. Acta Anthropologica Sinca. (04), 363-371. doi: 10.16359/j.cnki.cn11-1963/q.2009.04.006.

Zhao, M., Kong, Q.P., Wang, H.W., Peng, M.S., Xie, X.D., Wang, W.Z., et al. (2009). Mitochondrial genome evidence reveals successful Late Paleolithic settlement on the Tibetan Plateau. Proc Natl Acad Sci USA. 106(50), 21230-21235. doi: 10.1073/pnas.0907844106

Zhao, X. (2009) Physical anthropological and molecular archaeological research on ancient populations in Western Liaoning before Qin Dynasty. [dissertation/doctoral thesis]. [Changchun (Jilin)]: Jilin University

Zhou, J.T. (2012) The study of human skeletons and related archaeological issues in the early Longshan period in the Wuzhuangguoliang site in Jingbian, northern Shaanxi. [dissertation/master's thesis]. [Xi'an (Shaanxi)]: Northwest University

2 Supplementary Figures and Tables

2.1 Supplementary Figures

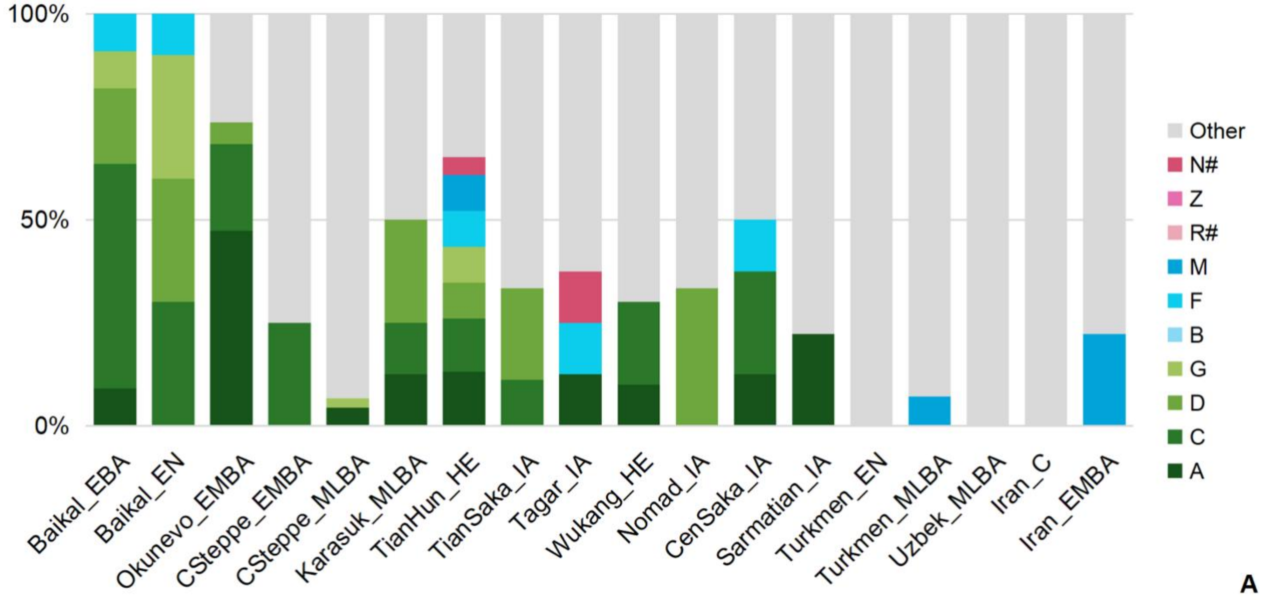

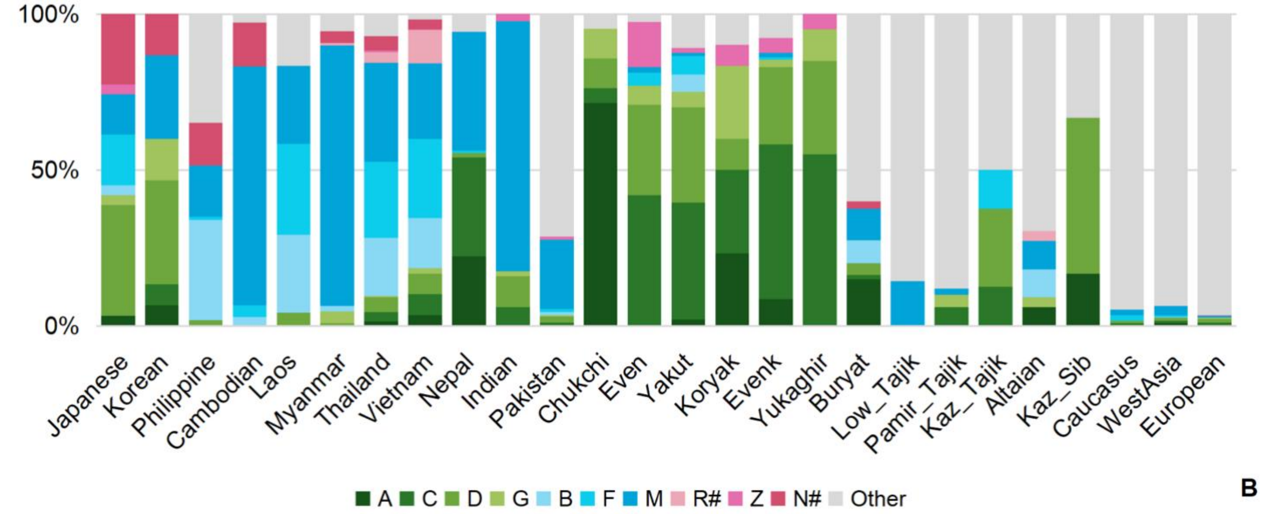

Supplementary Figure 1. Haplogroup frequency and haplogroup sharing analysis. **(A)** Haplogroup frequency of all ancient populations except for ancient populations in China. **(B)** Haplogroup frequency of all present-day populations. The haplogroups with green are those common in northeastern Asians (NEAs), and those with blue are common in southeastern Asians (SEAs). The haplogroups R# and N# represent the haplotypes shown in East Eurasians (such as haplotype R+16189, sub-haplogroups R11 and N9, which were observed in the Shimao-related populations). The other sub-haplogroups of R and N shown in West Eurasians were assigned to "others".

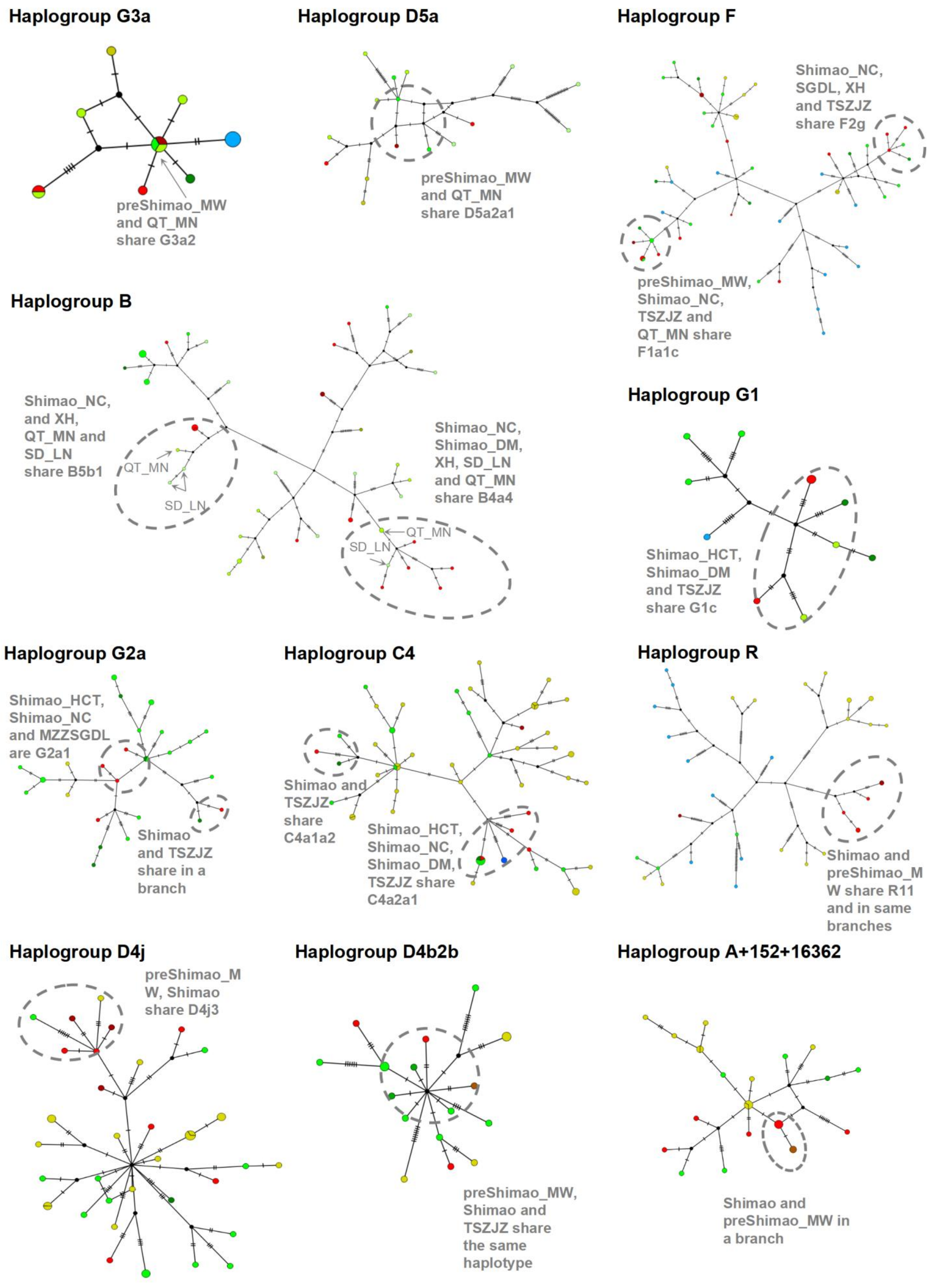
Haplogroup G3a
preShimao_MW
and QT_MN
share G3a2
Haplogroup D5a
preShimao_MW
and QT_MN
share D5a2a1
Haplogroup F
Shimao_NC,
SGDL, XH
and TSZJZ
share F2g
preShimao_MW,
Shimao_NC,
TSZJZ and
QT_MN share
F1a1c
Haplogroup B
Shimao_NC,
and XH,
QT_MN and
SD_LN
share B5b1
QT_MN
SD_LN
Shimao_NC,
Shimao_DM,
XH, SD_LN
and QT_MN
share B4a4
QT_MN
SD_LN
Haplogroup G1
Shimao_HCT,
Shimao_DM
and TSZJZ
share G1c
Haplogroup G2a
Shimao_HCT,
Shimao_NC
and MZZSGDL
are G2a1
Shimao
and TSZJZ
share in a
branch
Haplogroup C4
Shimao and
TSZJZ
share
C4a1a2
Shimao_HCT,
Shimao_NC,
Shimao_DM,
TSZJZ share
C4a2a1
Haplogroup R
Shimao and
preShimao_M
W share R11
and in same
branches
Haplogroup D4j
preShimao_M
W, Shimao
share D4j3
Haplogroup D4b2b
preShimao_MW,
Shimao and
TSZJZ share
the same
haplotype
Haplogroup A+152+16362
Shimao and
preShimao_MW in
a branch

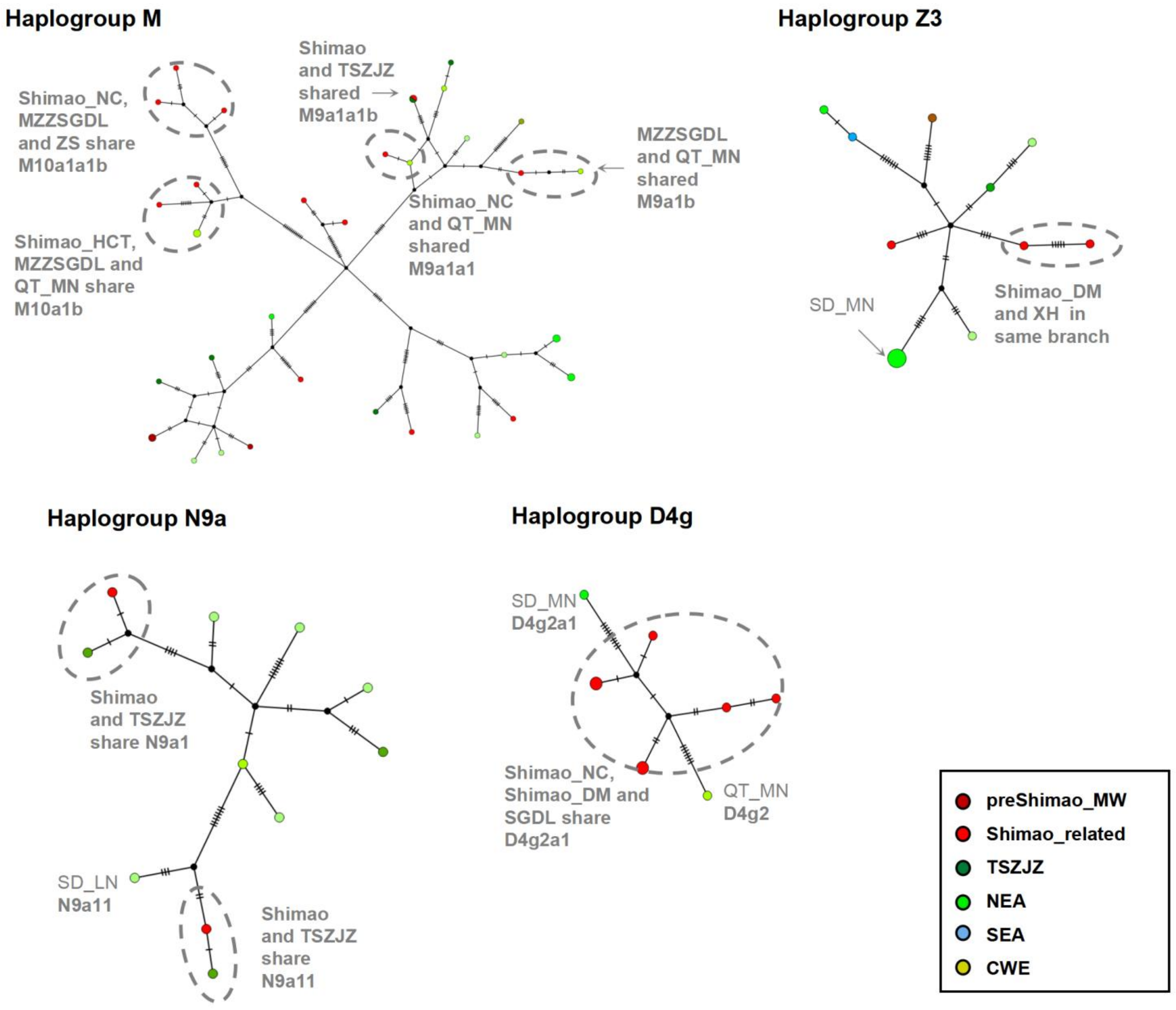

Supplementary Figure 2. The median-joining networks of haplotypes G3a2, D5a2a1 and F1a1c show the genetic connections between preShimao_MW and QT_MN**.** Haplotypes F1a1c, F2g, B4a4, B5b1**,** M9a1b, M9a1a1b, and M10a1b show the connections between Shimao-related populations and QT_MN. These haplotypes and haplotypes G2a1, Z3, R11, D4j3, A+152+16362, and D4g2a1 also show close genetic affinity in Shimao-related populations and with their preceding populations. Haplotypes G1c, G2a, C4a1a2 and C4a2a1, D4b2b, M9a1a1b, N9a and N9a11 are related to the ancient northern Chinese population TSZJZ. The size of the circles represents the proportion of each haplotype. The lengths of lines between nodes represent the number of mutations between two haplotypes. The different population groups are shown in different colors that are consistent with those groups in F_{ST} heatmap.

2.2 Supplementary Tables

Supplementary Table 1. The information of 172 new samples in this study.

Supplementary Table 2. The 801 ancient individuals from published papers.

Supplementary Table 3. The 7641 present-day individuals from published papers.

Supplementary Table 4. mtDNA haplogroup frequency of ancient samples in this study and different populations from Eurasia.

Supplementary Table 5. The haplogroup frequency of present-day populations from Eurasia.

Supplementary Table 6. The genetic distance and p value between Shimao-related populations and ancient and present-day population in this study.

Supplementary Table 7. The haplogroup sharing of ancient populations.

Supplementary Table 8. The haplogroup sharing between ancient YR populations and present-day populations in China.
